# The micro-eukaryotic community: An underrated component of the mammalian gut microbiota?

**DOI:** 10.3389/fmicb.2023.1123513

**Published:** 2023-03-16

**Authors:** Francisco Vargas-Albores, Estefanía Garibay-Valdez, Diana Medina-Félix, Marcel Martínez-Porchas

**Affiliations:** ^1^Centro de Investigación en Alimentación y Desarrollo, A.C. Biología de Organismos Acuáticos, Hermosillo, Sonora, Mexico; ^2^Departamento de Ecología, Universidad Estatal de Sonora, Hermosillo, Sonora, Mexico

**Keywords:** microbiota, micro-eukaryotic communities, gut eukaryome, quorum-sensing, host-associated microbes

The micro-eukaryotic community or “eukaryome” is defined as the fraction of microbes composed of nucleated organisms such as protists, fungi (filamentous fungi and yeasts), and metazoan parasites (cestodes, nematodes, and helminths) (Laforest-Lapointe and Arrieta, 2018). This diverse community has been largely overlooked in animal-microbiome studies in the last three decades compared to its prokaryotic counterpart. Here we argue why these organisms should be more carefully studied to approach microbiome studies from a multi-trophic perspective.

Molecular ecological surveys of animal gut microbiota have vastly focused on the prokaryotic fraction of the community, revealing a substantial bacterial diversity and vital functionality, whereas the eukaryotic composition has received less attention. The differential in attention between prokaryotes and eukaryotes is not unusual since prokaryotic communities are the most abundant, while eukaryotes represent between 2 and 5% of the microbial concentration (Scanlan and Marchesi, 2008). Other estimations calculated that in the mammalian gut microbiome, the fungal portion (mycobiome) constitutes 0.1% or less of the gut ecosystem (Nash et al., 2017; Zhai et al., 2020). However, from the cell and genome-size perspectives, the fungal fraction is not a marginal community as the fungal cell volume is hundreds of times bigger than bacterial cells volume, with genomes that can be tens or hundreds fold larger, representing significant biomass with vigorous production capacities of a diversity of biomolecules and metabolic power (Pettersen et al., 2022). For instance, in fungi, genomes vary from 9 to almost 180 megabases, encoding for approximately 10,000 to 25,000 genes, whereas bacterial genomes range from < 1 to 8 megabases encoding for 600 to 6,000 genes (Mohanta and Bae, 2015; Koduru, 2019).

Even though the information about bacterial and archaea commensals has increased significantly during the last decade (Jandhyala et al., 2015; Lin and Zhang, 2017) the comprehension of the gut microbiota as a multitrophic community has not advanced at the same pace because of the poor understanding of the diversity and functionality of other eukaryotic microorganisms thriving in the animal gut. In addition, designed microbiota consortia to be used in gnotobiotic murine models or as a therapeutic strategy are solely based on bacteria; such is the case of the Oligo-Mouse-Microbiota (Oligo-MM12), an *in vitro* designed microbiota based on members of the major bacterial phyla in the murine gut, and other designed prototypes to colonize the gut microbiota (Brugiroux et al., 2016; Lagkouvardos et al., 2016). Even though designed consortia can confer several benefits and restore functions in gnotobiotic models, it does not mimic the multitrophic reality. In addition, typical interaction approaches include a three-way microbial community interaction including commensal–pathogen, commensal–host, and pathogen–host interactions; however, if fungi and protozoan are included in the equation, a potential 55-way co-occurring interaction is obtained (Figure 1).

**Figure 1:**
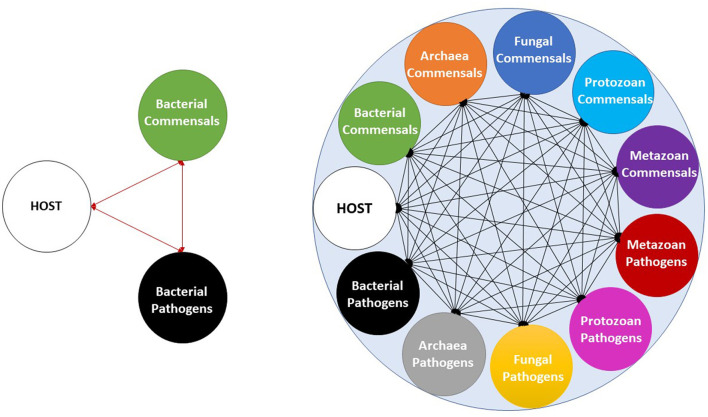
Illustration of gut microbial community interactions considering only bacteria (**left**; 3-way interaction), including archaea, fungi, protozoan, and metazoan (**right**; 55-way interaction). The illustration only shows the potential interactions occurring in the microbiota environment but does not indicate their frequency and intensity, nor if these are positive or negative.

The statistics derived from a simple search using the ISI Web of Knowledge platform with a pre-established search algorithm (gut microbiota OR intestinal microbiota OR gastrointestinal microbiota) showed that 97% of the last 210 scientific publications related to intestinal microbiota consider only prokaryotic microorganisms. Surprisingly, only 2.5% considered prokaryotes and microeukaryotes together, and 0.5% focused exclusively on microeukaryotes (Supplementary material 1). Whether this search could be modified to focus on microeukaryotes, using general terms like “microbiota” reveals the strong bias of the term to be associated exclusively with bacteria. Furthermore, in our search, we did not detect studies with gnotobiotic models to test eukaryotic microorganisms; however, it is true that these are minority fractions of the intestinal microbiota and are difficult to purify and cultivate, although this remains a crucial field for further research.

Despite microeukaryotes being usually associated with diseases, either by parasitic or pathogenic activities, evidence has demonstrated that they can provide commensal and beneficial species to the gut microbiota (Lukeš et al., 2015). For instance, metabolomics analyses and *in vivo* assays reported that the protozoan *Tritrichomonas musculis* could mechanistically influence the host glucose metabolism in a murine model by facilitating the production of a significant amount of free choline used by choline-utilizing bacteria, which is later transformed by the host to trimethylamine N-oxide as a final product, inducing hepatic gluconeogenesis (Kou et al., 2022). In addition, commensal mycobiota members can induce the host's immune response (Underhill and Iliev, 2014); as well, commensal protozoans are reported to enhance antibacterial defenses in murine models, increasing intestinal inflammation by triggering inflammasome activation in the gut epithelial cell (Chudnovskiy et al., 2016). Furthermore, Wei et al. (2020) intuited a cross-talking activity between protozoans and bacteria and detected a balance maintained by three elements: bacteria, protozoans, and dietary nutrients. In this regard, certain protozoans in the animal gut seem to favor bacterial diversity and, ultimately, the host's health. For instance, Audebert et al. (2016) demonstrated that Blastocystis, a typical single-celled eukaryote in the human gut microbiota, induces a higher bacterial diversity in the fecal microbiota of Blastocystis-colonized patients compared to those without this protist, and concluded that Blastocystis colonization might contribute to a healthy gut microbiota rather than causing dysbiosis. Therefore, the gut eukaryotes contribute to an ecological balance of the microbiota, necessary to maintain the health of the host.

Perhaps one of the most evident examples of micro-eukaryotes contribution to gut microbiota involves the ubiquitous yeast Saccharomyces, several of which have been used as probiotics or paraprobiotics. For example, *S. boulardii* exerts beneficial luminal and trophic actions within the gut microbiota of adult humans (McFarland, 2010). The luminal action includes an antitoxic effect against toxins secreted by pathogens like *Clostrodium difficile, Escherlichia coli*, and the Cholera toxin; it also has antimicrobial activity, modulation of intestinal microbiota, and metabolic activity producing short-chain fatty acids favoring the colonic function. Regarding the trophic action of *S. boulardii*, it produces polyamines that favor enterocyte maturation and increase disaccharide levels which are beneficial in viral diarrhea; its presence also enhances immunoglobulin A levels. Finally, *S. boulardii* has an anti-inflammatory effect by cross-talking through molecular signals and decreasing the synthesis of inflammatory cytokines. The clinical use of yeast is recommended to prevent antibiotic-associated diarrhea and other kinds of diarrhea, *Helicobacter pylori* symptoms, *Clostridium* infections, inflammatory bowel disease, irritable bowel syndrome, giardiasis, and other conditions (McFarland, 2010).

Eukaryotes (including opportunistic) at mucosal surfaces are controlled by the normal microbiota, the epithelium, and its innate immune system; these are also regulated by a continuous or transient cross-talk between the eukaryote and the host immune system while maintaining homeostasis with resident microbial populations, ensuring the balance between tolerogenic and proinflammatory responses (Rizzetto et al., 2015).

The gut eukaryotes are assumed to be in constant communication with the host and the gut prokaryotes. Quorum sensing (QS) signal molecules produced by prokaryotic cells is not restricted to bacterial communication since allowing interkingdom communication with eukaryotic cells (mammalian, plant, and fungi cells) (Fan et al., 2022). The QS signaling system from prokaryotic cells includes the N-acyl-L-homoserine lactones (AHLs), autoinducer-2 (AI-2), and auto-inducible peptides (AIPs), which regulates the interkingdom communication. Eukaryotic animal cells possess AHLs receptors that sense bacterial signals, such as the AhR receptor that binds to 3-oxo-C12-HSL in the cytoplasm and then transfers into the nucleus to regulate host immunity. In addition, eukaryotic signals like hormones, neurotransmitters, or immune system molecules have been shown to modulate bacterial physiology (biofilm formation, growth, chemotaxis, and potential adhesion). Likewise, bacteria possess QseC sensor Kinase, a receptor to sense the host hormones (Norepinephrine/Epinephrine) (Boukerb et al., 2021). In prokaryotes and fungi, QS communication the MHF [4-hydroxy-5-methyl furan-3 (2H)-one] production by fungi cells has been reported, which is catalyzed and produced by the Cff1p protein and sensed by the AI-2 receptor LuxP to regulate the QS regulatory network, this last prokaryotic-eukaryotic interaction is the less elucidated (Fan et al., 2022).

Similarly, the eukaryotic-host QS signaling system is poorly studied, despite the several QS molecules produced by fungal communities such as pheromones, farnesol, tyrosol, and oxylipins, among others (Mehmood et al., 2019). There is relatively scarce information about the direct interactions between microeukaryotes and host cells. Consequently, the complex gut eukaryotes-host interactions are not yet elucidated but offer a notion of a balance of microbial communities through molecular means of communication. In addition, interkingdom interactions between commensal microeukaryotes and the bacterial community are intuited but remain an almost unexplored field.

Like its prokaryotic counterpart, the gut eukaryotic structure and composition are influenced by a variety of factors such as diet, age, nutritional and physiological condition, disease, antimicrobials, geography, and others (Hamad et al., 2016; Wheeler et al., 2016; Ahmad et al., 2020; Ramayo-Caldas et al., 2020). In the case of antimicrobials, the gut eukaryotes can be directly (antiparasitic and antifungals) or indirectly affected (antibiotics); despite antibiotics being designed to eradicate bacteria, the imbalance of the prokaryotic community may influence the eukaryotic counterpart; for example, antibiotics induce bacterial dysbiosis, altering the taxonomic profile of these prokaryotes leading to fungal overgrowth (Laforest-Lapointe and Arrieta, 2018). Therefore, as in the case of bacteria, several gut microeukaryotes are harmless but beneficial under optimal balancing conditions, and it is the imbalance of these microbial communities that provides the scenario for some pathobionts to emerge. Finally, the term dysbiosis, which in most studies is markedly associated with affectations in bacterial communities, should encompass a multi-kingdom perspective; in this regard, gut microbiota studies dealing with dysbiosis should highlight the specificity of the kingdom in which the approach focuses. In the end, the micro-eukaryotic community is still an underrated component of the animal gut microbiota but, as described here, can fill several information gaps that persist, particularly at the equilibrium level of multitrophic communities, the contribution of microeukaryotes to the health and physiology of the host and inter-kingdom communication systems. However, to obtain much of the missing knowledge, it is necessary to delve deeper into the omics sciences, since, up to now, most studies use targeted metagenomics; that is, biomarkers such as 16S and 18S ribosomal RNA, and the internal transcribed spacer (ITS). Metagenomics and meta-transcriptomics could provide more robust and precise information regarding the functional capabilities, contributions of and responses of the eukaryotic fractions under diverse scenarios, but bioinformatics pipelines should be improved to differentiate this group from the rest of the microbes.

## Author contributions

FV-A and MM-P contributed to the conception and design of the article. DM-F and MM-P organized the database. FV-A, EG-V, DM-F, and MM-P wrote sections of the manuscript. All authors approved the final version of the editorial.
