# Supplementary material for: The micro-eukaryotic community: An underrated component of the mammalian gut microbiota?
Source: Front Microbiol. 2023 Mar 16;14:1123513. doi: 10.3389/fmicb.2023.1123513 (PMC10060968; doi:10.3389/fmicb.2023.1123513)
Supplement: Supplementary file 1 [file Data_Sheet_1.doc]

Supplementary Material

**The micro-eukaryotic community: an underrated component of the animal gut microbiota?**

**Francisco Vargas-Albores^1^, Estefanía Garibay-Valdez^1^, Diana Medina-Félix^2^, Marcel Martínez-Porchas^1*^**

^1^Centro de Investigación en Alimentación y Desarrollo, A.C. Biología de Organismos Acuáticos. Sonora, México.

^2^Universidad Estatal de Sonora. Unidad Hermosillo. Sonora, México.

*** Correspondence:**Marcel Martínez-Porchas
marcel@ciad.mx

**1. Supplementary Table**

**Table S1**. List of scientific articles derived from the search using the ISI Web of Knowledge platform with a pre-established search algorithm (gut microbiota OR intestinal microbiota OR gastrointestinal microbiota).

|  | **Author** | | **Year** | **Research Title** | **Gut microbiota** | **Marker Gen** | **Host** | **DOI link** |
| --- | --- | --- | --- | --- | --- | --- | --- | --- |
| 1 | | Deng, LJ; Zhou, XY; Lan, ZF; Tang, KR; Zhu, XX; Mo, XW; Zhao, ZY; Zhao, ZQ; Wu, MS | 2022 | Simotang Alleviates the Gastrointestinal Side Effects of Chemotherapy by Altering Gut Microbiota | **Prokaryote** | 16S | Mice | <http://dx.doi.org/10.4014/jmb.2110.10018> |
| 2 | | You, WD; Zhu, YR; Wei, AQ; Du, J; Wang, YD; Zheng, PD; Tu, MD; Wang, H; Wen, L; Yang, XF | 2022 | Traumatic Brain Injury Induces Gastrointestinal Dysfunction and Dysbiosis of Gut Microbiota Accompanied by Alterations of Bile Acid Profile | **Prokaryote** | 16S | Mice | <http://dx.doi.org/10.1089/neu.2020.7526> |
| 3 | | Sugden, S; St Clair, CC; Stein, LY | 2021 | Individual and Site-Specific Variation in a Biogeographical Profile of the Coyote Gastrointestinal Microbiota | **Prokaryote** | 16S | Wild coyote | <http://dx.doi.org/10.1007/s00248-020-01547-0> |
| 4 | | Zhang, BB; Wu, XY; Song, QY; Ning, A; Liang, JY; Song, LG; Liu, JH; Zhang, YS; Yuan, DJ; Sun, X; Wu, ZD | 2020 | Gut Microbiota Modulates Intestinal Pathological Injury in Schistosoma japonicum-Infected Mice | **Prokaryote** | 16S | Mice | <http://dx.doi.org/10.3389/fmed.2020.588928> |
| 5 | | Qi, YZ; Jiang, YH; Jiang, LY; Shao, LL; Yang, XS; Yang, CH | 2021 | An Insight Into Intestinal Microbiota of Spontaneously Hypertensive Rats After Valsartan Administration | **Prokaryote** | 16S | Rats | <http://dx.doi.org/10.1177/15593258211011342> |
| 6 | | Wang, YH; Shi, Y; Li, WH; Wang, S; Zheng, JY; Xu, GH; Li, GX; Shen, XF; Yang, JJ | 2022 | Gut microbiota imbalance mediates intestinal barrier damage in high-altitude exposed mice | **Prokaryote** | 16S | Mice | <http://dx.doi.org/10.1111/febs.16409> |
| 7 | | Chaves, LD; McSkimming, DI; Bryniarski, MA; Honan, AM; Abyad, S; Thomas, SA; Wells, S; Buck, M; Sun, YJ; Genco, RJ; Quigg, RJ; Yacoub, R | 2018 | Chronic kidney disease, uremic milieu, and its effects on gut bacterial microbiota dysbiosis | **Prokaryote** | 16S | Mice | <http://dx.doi.org/10.1152/ajprenal.00092.2018> |
| 8 | | Lee, SM; Kim, N; Park, JH; Nam, RH; Yoon, K; Lee, DH | 2018 | Comparative Analysis of Ileal and Cecal Microbiota in Aged Rats | **Prokaryote** | 16S | Rats | <http://dx.doi.org/10.15430/JCP.2018.23.2.70> |
| 9 | | Li, N; Zuo, B; Huang, SM; Zeng, BH; Han, DD; Li, TT; Liu, T; Wu, ZH; Wei, H; Zhao, JC; Wang, JJ | 2020 | Spatial heterogeneity of bacterial colonization across different gut segments following inter-species microbiota transplantation | **Prokaryote** | 16S | Mice | <http://dx.doi.org/10.1186/s40168-020-00917-7> |
| 10 | | Zhang, QY; Cheng, L; Wang, JJ; Hao, MZ; Che, HL | 2021 | Antibiotic-Induced Gut Microbiota Dysbiosis Damages the Intestinal Barrier, Increasing Food Allergy in Adult Mice | **Prokaryote** | 16S | Mice | <http://dx.doi.org/10.3390/nu13103315> |
| 11 | | Bhattarai, Y; Si, J; Pu, M; Ross, OA; McLean, PJ; Till, L; Moor, W; Grover, M; Kandimalla, KK; Margolis, KG; Farrugia, G; Kashyap, PC | 2021 | Role of gut microbiota in regulating gastrointestinal dysfunction and motor symptoms in a mouse model of Parkinson's disease. | **Prokaryote** | 16S | Mice | <http://dx.doi.org/10.1080/19490976.2020.1866974> |
| 12 | | Gu, M; Samuelson, DR; de la Rua, NM; Charles, TP; Taylor, CM; Luo, M; Siggins, RW; Shellito, JE; Welsh, DA | 2022 | Host innate and adaptive immunity shapes the gut microbiota biogeography | **Prokaryote** | 16S | Mice | <http://dx.doi.org/10.1111/1348-0421.12963> |
| 13 | | Wang, HQ; Huang, J; Ding, YA; Zhou, JW; Gao, GZ; Han, H; Zhou, JR; Ke, LJ; Rao, PF; Chen, TB; Zhang, LX | 2022 | Nanoparticles Isolated From Porcine Bone Soup Ameliorated Dextran Sulfate Sodium-Induced Colitis and Regulated Gut Microbiota in Mice | **Prokaryote** | 16S | Mice | <http://dx.doi.org/10.3389/fnut.2022.821404> |
| 14 | | Zhao, BY; Zhou, BL; Dong, CY; Zhang, R; Xie, DY; Tian, YM; Yang, L | 2021 | Lactobacillus reuteri Alleviates Gastrointestinal Toxicity of Rituximab by Regulating the Proinflammatory T Cells in vivo | **Prokaryote** | 16S | Mice | <http://dx.doi.org/10.3389/fmicb.2021.645500> |
| 15 | | Rao, JJ; Xie, RN; Lin, L; Jiang, J; Du, L; Zeng, XD; Li, GY; Wang, CM; Qiao, Y | 2021 | Fecal microbiota transplantation ameliorates gut microbiota imbalance and intestinal barrier damage in rats with stress-induced depressive-like behavior | **Prokaryote** | 16S | Rats | <http://dx.doi.org/10.1111/ejn.15192> |
| 16 | | Li, YY; Wang, YC; Shi, F; Zhang, XJ; Zhang, YT; Bi, KF; Chen, XQ; Li, LJ; Diao, HY | 2022 | Phospholipid metabolites of the gut microbiota promote hypoxia-induced intestinal injury via CD1d-dependent gamma delta T cells | **Prokaryote** | 16S | Mice | <http://dx.doi.org/10.1080/19490976.2022.2096994> |
| 17 | | Daly, K; Kelly, J; Moran, AW; Bristow, R; Young, IS; Cossins, AR; Bravo, D; Shirazi-Beechey, SP | 2019 | Host selectively contributes to shaping intestinal microbiota of carnivorous and omnivorous fish | **Prokaryote** | 16S | Fish | <http://dx.doi.org/10.2323/jgam.2018.07.003> |
| 18 | | Yang, JQ; Xiong, P; Bai, L; Zhang, ZY; Zhou, Y; Chen, C; Xie, ZR; Xu, Y; Chen, MH; Wang, HW; Zhu, M; Yu, JH; Wang, KH | 2021 | The Association of Altered Gut Microbiota and Intestinal Mucosal Barrier Integrity in Mice With Heroin Dependence | **Prokaryote** | 16S | Mice | <http://dx.doi.org/10.3389/fnut.2021.765414> |
| 19 | | Ma, ZJ; Wang, HJ; Ma, XJ; Li, Y; Yang, HJ; Li, H; Su, JR; Zhang, CE; Huang, LQ | 2020 | Modulation of gut microbiota and intestinal barrier function during alleviation of antibiotic-associated diarrhea with Rhizoma Zingiber officinale (Ginger) extract | **Prokaryote** | 16S | Rats | <http://dx.doi.org/10.1039/d0fo01536a> |
| 20 | | Chen, WH; Yu, LL; Zhu, B; Qin, LP | 2022 | Dendrobium officinale Endophytes May Colonize the Intestinal Tract and Regulate Gut Microbiota in Mice | **Prokaryote** | 16S | Mice | <http://dx.doi.org/10.1155/2022/2607506> |
| 21 | | Li, AY; Yang, Y; Zhang, Y; Lv, SJ; Jin, TH; Li, K; Han, ZQ; Li, YZ | 2021 | Microbiome analysis reveals the alterations in gut microbiota in different intestinal segments of Yimeng black goats | **Prokaryote** | 16S | Goats | <http://dx.doi.org/10.1016/j.micpath.2021.104900> |
| 22 | | Sarhadi, V; Mathew, B; Kokkola, A; Karla, T; Tikkanen, M; Rautelin, H; Lahti, L; Puolakkainen, P; Knuutila, S | 2021 | Gut microbiota of patients with different subtypes of gastric cancer and gastrointestinal stromal tumors | **Prokaryote** | 16S | Human | <http://dx.doi.org/10.1186/s13099-021-00403-x> |
| 23 | | Geng, SJ; Cheng, SS; Li, Y; Wen, ZS; Ma, X; Jiang, XM; Wang, YZ; Han, XY | 2018 | Faecal Microbiota Transplantation Reduces Susceptibility to Epithelial Injury and Modulates Tryptophan Metabolism of the Microbial Community in a Piglet Model | **Prokaryote** | 16S | Pig | <http://dx.doi.org/10.1093/ecco-jcc/jjy103> |
| 24 | | Dong, ZH; Zhang, SMY; Cheng, YW; He, XC; Haase, I; Liang, Y; Jiang, Y; Wu, YJ | 2022 | Comparative analysis of the intestinal tract microbiota and feeding habits of five sympatric flycatchers | **Prokaryote** | 16S | Birds | <http://dx.doi.org/10.1016/j.avrs.2022.100050> |
| 25 | | Yan, YP; Wang, JF; Qiu, S; Duan, YC; Si, W | 2022 | The Lumenal Microbiota Varies Biogeographically in the Gastrointestinal Tract of Rhesus Macaques | **Prokaryote** | 16S | Monkeys | <http://dx.doi.org/10.1128/spectrum.00343-22> |
| 26 | | Lucarini, E; Di Pilato, V; Parisio, C; Micheli, L; Toti, A; Pacini, A; Bartolucci, G; Baldi, S; Niccolai, E; Amedei, A; Rossolini, GM; Nicoletti, C; Cryan, JF; O'Mahony, SM; Ghelardini, C; Mannelli, LD | 2022 | Visceral sensitivity modulation by faecal microbiota transplantation: the active role of gut bacteria in pain persistence | **Prokaryote** | 16S | Rats | <http://dx.doi.org/10.1097/j.pain.0000000000002438> |
| 27 | | Cussotto, S; Strain, CR; Fouhy, F; Strain, RG; Peterson, VL; Clarke, G; Stanton, C; Dinan, TG; Cryan, JF | 2019 | Differential effects of psychotropic drugs on microbiome composition and gastrointestinal function | **Prokaryote** | 16S | Rats | <http://dx.doi.org/10.1007/s00213-018-5006-5> |
| 28 | | Mao, TY; Su, CW; Ji, QR; Chen, CY; Wang, RJ; Kumar, DV; Lan, JG; Jiao, LF; Shi, HN | 2021 | Hyaluronan-induced alterations of the gut microbiome protects mice against Citrobacter rodentium infection and intestinal inflammation | **Prokaryote** | 16S | Mice | <http://dx.doi.org/10.1080/19490976.2021.1972757> |
| 29 | | Marsh, R; Gavillet, H; Hanson, L; Ng, C; Mitchell-Whyte, M; Major, G; Smyth, AR; Rivett, D; van der Gast, C | 2022 | Intestinal function and transit associate with gut microbiota dysbiosis in cystic fibrosis | **Prokaryote** | 16S | Human | <http://dx.doi.org/10.1016/j.jcf.2021.11.014> |
| 30 | | Wang, YB; Xu, LP; Sun, XL; Wan, XH; Sun, GR; Jiang, RR; Li, WT; Tian, YD; Liu, XJ; Kang, XT | 2020 | Characteristics of the fecal microbiota of high- and low-yield hens and effects of fecal microbiota transplantation on egg production performance | **Prokaryote** | 16S | Birds | <http://dx.doi.org/10.1016/j.rvsc.2020.01.020> |
| 31 | | van Kessel, SP; Bullock, A; van Dijk, G; El Aidy, S | 2022 | Parkinson's Disease Medication Alters Small Intestinal Motility and Microbiota Composition in Healthy Rats | **Prokaryote** | 16S | Rats | <https://journals.asm.org/doi/full/10.1128/msystems.01191-21> |
| 32 | | Ren, XX; Liu, L; Liu, PK; Gamallat, Y; Xin, Y; Shang, D | 2018 | Polysaccharide extracted from Enteromorpha ameliorates Cisplastin-induced small intestine injury in mice | **Prokaryote** | 16S | Mice | <http://dx.doi.org/10.1016/j.jff.2018.08.023> |
| 33 | | Zhang, CY; Peng, XX; Shao, HQ; Li, XY; Wu, Y; Tan, ZJ | 2021 | Gut Microbiota Comparison Between Intestinal Contents and Mucosa in Mice With Repeated Stress-Related Diarrhea Provides Novel Insight | **Prokaryote** | 16S | Mice | <http://dx.doi.org/10.3389/fmicb.2021.626691> |
| 34 | | Zhang, Z; Yu, YX; Jiang, Y; Wang, YG; Liao, MJ; Rong, XJ; Liu, Q | 2020 | The intestine of artificially bred larval turbot (Scophthalmus maximus) contains a stable core group of microbiota | **Prokaryote** | 16S | Fish | <http://dx.doi.org/10.1007/s00203-020-01984-y> |
| 35 | | Li, X; Li, ZC; Chang, YX; Hou, FY; Huang, ZY; Ni, H; Yang, RF; Bi, YJ | 2018 | Successful transplantation of guinea pig gut microbiota in mice and its effect on pneumonic plague sensitivity | **Prokaryote** | 16S | Mice and pigs | <http://dx.doi.org/10.7717/peerj.5637> |
| 36 | | Bai, JZ; Cai, YJ; Huang, ZY; Gu, YQ; Huang, NA; Sun, R; Zhang, GM; Liu, RP | 2022 | Shouhui Tongbian Capsule ameliorates constipation via gut microbiota-5-HT-intestinal motility axis | **Prokaryote** | 16S | Mice | <http://dx.doi.org/10.1016/j.biopha.2022.113627> |
| 37 | | White, EC; Houlden, A; Bancroft, AJ; Hayes, KS; Goldrick, M; Grencis, RK; Roberts, IS | 2018 | Manipulation of host and parasite microbiotas: Survival strategies during chronic nematode infection | **Prokaryote** | 16S | Mice | <http://dx.doi.org/10.1126/sciadv.aap7399> |
| 38 | | Wu, CC; Tung, YT; Chen, SY; Lee, WT; Lin, HT; Yen, GC | 2020 | Anti-Inflammatory, Antioxidant, and Microbiota-Modulating Effects of Camellia Oil from Camellia brevistyla on Acetic Acid-Induced Colitis in Rats | **Prokaryote** | 16S | Mice | <http://dx.doi.org/10.3390/antiox9010058> |
| 39 | | Guo, SJ; Jiang, DJ; Zhang, Q; Zhang, Y; Yao, WF; Cao, YD; Bao, BH; Tang, YP; Kang, A; Zhang, L | 2021 | Diverse role of gut microbiota on reduction of ascites and intestinal injury in malignant ascites effusion rats treated with Euphorbia kansui stir-fried with vinegar | **Prokaryote** | 16S | Rats | <http://dx.doi.org/10.1016/j.jep.2020.113489> |
| 40 | | Su, SF; Zhao, YP; Liu, ZZ; Liu, GQ; Du, M; Wu, J; Bai, DY; Li, B; Bou, G; Zhang, XZ; Dugarjaviin, M | 2020 | Characterization and comparison of the bacterial microbiota in different gastrointestinal tract compartments of Mongolian horses | **Prokaryote** | 16S | Horse | <http://dx.doi.org/10.1002/mbo3.1020> |
| 41 | | Yeung, CY; Chiau, JSC; Cheng, ML; Chan, WT; Chang, SW; Chang, YH; Jiang, CB; Lee, HC | 2020 | Modulations of probiotics on gut microbiota in a 5-fluorouracil-induced mouse model of mucositis | **Prokaryote** | 16S | Mice | <http://dx.doi.org/10.1111/jgh.14890> |
| 42 | | Gao, H; Zhang, WC; Wu, ZY; Wang, HY; Hui, AL; Meng, L; Chen, PP; Xian, ZJ; He, YW; Li, HH; Du, B; Zhang, HW | 2018 | Preparation, characterization and improvement in intestinal function of polysaccharide fractions from okra | **Prokaryote** | 16S | Mice | <http://dx.doi.org/10.1016/j.jff.2018.09.035> |
| 43 | | Gai, XW; Wang, HW; Li, YQ; Zhao, HT; He, C; Wang, ZH; Zhao, HL | 2021 | Fecal Microbiota Transplantation Protects the Intestinal Mucosal Barrier by Reconstructing the Gut Microbiota in a Murine Model of Sepsis | **Prokaryote** | 16S | Mice | <http://dx.doi.org/10.3389/fcimb.2021.736204> |
| 44 | | Cirstea, MS; Yu, AC; Golz, E; Sundvick, K; Kliger, D; Radisavljevic, N; Foulger, LH; Mackenzie, M; Huan, T; Finlay, BB; Appel-Cresswell, S | 2020 | Microbiota Composition and Metabolism Are Associated With Gut Function in Parkinson's Disease | **Prokaryote** | 16S | Human | <http://dx.doi.org/10.1002/mds.28052> |
| 45 | | Miranda-Ribera, A; Serena, G; Liu, JD; Fasano, A; Kingsbury, MA; Fiorentino, MR | 2022 | The Zonulin-transgenic mouse displays behavioral alterations ameliorated via depletion of the gut microbiota | **Prokaryote** | 16S | Mice | <http://dx.doi.org/10.1080/21688370.2021.2000299> |
| 46 | | Rzeznitzeck, J; Hoerr, FJ; Rychlik, I; Methling, K; Lalk, M; Rath, A; von Altrock, A; Rautenschlein, S | 2022 | Morphology, microbiota, and metabolome along the intestinal tract of female turkeys | **Prokaryote** | 16S | Birds | <http://dx.doi.org/10.1016/j.psj.2022.102046> |
| 47 | | Skalski, JH; Limon, JJ; Sharma, P; Gargus, MD; Nguyen, C; Tang, J; Coelho, AL; Hogaboam, CM; Crother, TR; Underhill, DM | 2018 | Expansion of commensal fungus Wallemia mellicola in the gastrointestinal mycobiota enhances the severity of allergic airway disease in mice | **Eukaryote + Prokaryote** | ITS + 16S | Mice | <http://dx.doi.org/10.1371/journal.ppat.1007260> |
| 48 | | Tong, AJ; Hu, RK; Wu, LX; Lv, XC; Li, X; Zhao, LN; Liu, B | 2020 | Ganoderma polysaccharide and chitosan synergistically ameliorate lipid metabolic disorders and modulate gut microbiota composition in high fat diet-fed golden hamsters | **Prokaryote** | 16S | Hamsters | <http://dx.doi.org/10.1111/jfbc.13109> |
| 49 | | Li, XJ; Guo, R; Wu, XJ; Liu, X; Ai, LZ; Sheng, Y; Song, ZB; Wu, Y | 2020 | Dynamic digestion of tamarind seed polysaccharide: Indigestibility in gastrointestinal simulations and gut microbiota changes in vitro | **Prokaryote** | 16S | Culture (in vitro) | <http://dx.doi.org/10.1016/j.carbpol.2020.116194> |
| 50 | | Palombo, G; Merone, M; Altomare, A; Gori, M; Terradura, C; Bacco, L; Del Chierico, F; Putignani, L; Cicala, M; Guarino, MPL; Piemonte, V | 2021 | The impact of the intestinal microbiota and the mucosal permeability on three different antibiotic drugs | **Prokaryote** | 16S | Human | <http://dx.doi.org/10.1016/j.ejps.2021.105869> |
| 51 | | Wu, ZB; Zhang, QQ; Lin, YY; Hao, JW; Wang, SY; Zhang, JY; Li, AH | 2021 | Taxonomic and Functional Characteristics of the Gill and Gastrointestinal Microbiota and Its Correlation with Intestinal Metabolites in NEW GIFT Strain of Farmed Adult Nile Tilapia (Oreochromis niloticus) | **Prokaryote** | 16S | Fish | <http://dx.doi.org/10.3390/microorganisms9030617> |
| 52 | | Li, ZT; Wang, JW; Hu, XH; Zhu, L; Jiang, Y; Gao, MJ; Zhan, XB | 2022 | The effects of high-fat foods on gut microbiota and small molecule intestinal gases: release kinetics and distribution in vitro colon model | **Prokaryote** | 16S | in vitro | <http://dx.doi.org/10.1016/j.heliyon.2022.e10911> |
| 53 | | Li, LY; Wang, Q; Gao, YY; Liu, L; Duan, YJ; Mao, DQ; Luo, Y | 2021 | Colistin and amoxicillin combinatorial exposure alters the human intestinal microbiota and antibiotic resistome in the simulated human intestinal microbiota | **Prokaryote** | 16S | Culture (in vitro) | <http://dx.doi.org/10.1016/j.scitotenv.2020.141415> |
| 54 | | Zhou, YL; Zhang, F; Mao, LQ; Feng, TF; Wang, KJ; Xu, MS; Lv, B; Wang, X |  | Bifico relieves irritable bowel syndrome by regulating gut microbiota dysbiosis and inflammatory cytokines | **Prokaryote** | 16S | Mice | <http://dx.doi.org/10.1007/s00394-022-02958-0> |
| 55 | | Roubaud-Baudron, C; Ruiz, VE; Swan, AM; Vallance, BA; Ozkul, C; Pei, ZH; Li, J; Battaglia, TW; Perez-Perez, GI; Blaser, MJ | 2019 | Long-Term Effects of Early-Life Antibiotic Exposure on Resistance to Subsequent Bacterial Infection | **Prokaryote** | 16S | Mice | <http://dx.doi.org/10.1128/mBio.02820-19> |
| 56 | | Xu, GL; Xing, W; Li, TL; Xue, M; Ma, ZH; Jiang, N; Luo, L | 2019 | Comparative study on the effects of different feeding habits and diets on intestinal microbiota in Acipenser baeri Brandt and Huso huso | **Prokaryote** | 16S | Fish | <http://dx.doi.org/10.1186/s12866-019-1673-6> |
| 57 | | He, SY; Cui, SD; Song, W; Jiang, YH; Chen, HS; Liao, DJ; Lu, XP; Li, J; Chen, XQ; Peng, L | 2022 | Interleukin-17 Weakens the NAFLD/NASH Process by Facilitating Intestinal Barrier Restoration Depending on the Gut Microbiota | **Prokaryote** | 16S | Mice | <http://dx.doi.org/10.1128/mbio.03688-21> |
| 58 | | Pan, GH; Liu, BD; Li, SX; Han, ML; Gao, L; Xu, GH; Du, Q; Xie, LW | 2020 | Kuijieling, a Chinese medicine alleviates DSS-induced colitis in C57BL/6Jmouse by improving the diversity and function of gut microbiota | **Prokaryote** | 16S | Mice | <http://dx.doi.org/10.1093/femsle/fnaa082> |
| 59 | | Nagasue, T; Hirano, A; Torisu, T; Umeno, J; Shibata, H; Moriyama, T; Kawasaki, K; Fujioka, S; Fuyuno, Y; Matsuno, Y; Esaki, M; Kitazono, T | 2022 | The Compositional Structure of the Small Intestinal Microbial Community via Balloon-Assisted Enteroscopy | **Prokaryote** | 16S | Human | <http://dx.doi.org/10.1159/000524023> |
| 60 | | Xu, HH; Cao, JF; Li, XY; Lu, XC; Xia, Y; Fan, DP; Zhao, HY; Ju, DH; Xiao, C | 2020 | Regional Differences in the Gut Microbiota and Gut-Associated Immunologic Factors in the Ileum and Cecum of Rats With Collagen-Induced Arthritis | **Prokaryote** | 16S | Rats | <http://dx.doi.org/10.3389/fphar.2020.587534> |
| 61 | | Wei, J; Guo, XW; Liu, H; Chen, YY; Wang, WM | 2018 | The variation profile of intestinal microbiota in blunt snout bream (Megalobrama amblycephala) during feeding habit transition | **Prokaryote** | 16S | Fish | <http://dx.doi.org/10.1186/s12866-018-1246-0> |
| 62 | | Stefura, T; Zapala, B; Stoj, A; Gosiewski, T; Skomarovska, O; Krzysztofik, M; Pedziwiatr, M; Major, P | 2020 | Does Postoperative Oral and Intestinal Microbiota Correlate with the Weight-Loss Following Bariatric Surgery?-A Cohort Study | **Prokaryote** | 16S | Human | <http://dx.doi.org/10.3390/jcm9123863> |
| 63 | | Arita, S; Inagaki-Ohara, K | 2019 | High-fat-diet-induced modulations of leptin signaling and gastric microbiota drive precancerous lesions in the stomach | **Prokaryote** | 16S | Mice | <http://dx.doi.org/10.1016/j.nut.2019.110556> |
| 64 | | Stefura, T; Zapata, B; Gosiewski, T; Krzysztofik, M; Skomarovska, O; Major, P | 2021 | Relationship between bariatric surgery outcomes and the preoperative gastrointestinal microbiota: a cohort study | **Prokaryote** | 16S | Human | <http://dx.doi.org/10.1016/j.soard.2021.01.011> |
| 65 | | Yang, GL; Zhang, SH; Li, ZQ; Huang, J; Liu, Y; Liu, Y; Wang, QK; Li, XY; Yan, YF; Li, M | 2020 | Comparison Between the Gut Microbiota in Different Gastrointestinal Segments of Large-Tailed Han and Small-Tailed Han Sheep Breeds with High-Throughput Sequencing | **Prokaryote** | 16S | Sheep | <http://dx.doi.org/10.1007/s12088-020-00885-4> |
| 66 | | Huang, XY; Fang, Q; Rao, T; Zhou, LP; Zeng, XC; Tan, ZR; Chen, LL; Ouyang, DS | 2020 | Leucovorin ameliorated methotrexate induced intestinal toxicity via modulation of the gut microbiota | **Prokaryote** | 16S | Mice | <http://dx.doi.org/10.1016/j.taap.2020.114900> |
| 67 | | Ji, SK; Jiang, T; Yan, H; Guo, CY; Liu, JJ; Su, HW; Alugongo, GM; Shi, HT; Wang, YJ; Cao, ZJ; Li, SL | 2018 | Ecological Restoration of Antibiotic-Disturbed Gastrointestinal Microbiota in Foregut and Hindgut of Cows | **Prokaryote** | 16S | Cows | <http://dx.doi.org/10.3389/fcimb.2018.00079> |
| 68 | | Zhuang, XJ; Tian, ZY; Li, L; Zeng, ZR; Chen, MH; Xiong, LS | 2018 | Fecal Microbiota Alterations Associated With Diarrhea-Predominant Irritable Bowel Syndrome | **Prokaryote** | 16S | Human | <http://dx.doi.org/10.3389/fmicb.2018.01600> |
| 69 | | Le, D; Nguyen, P; Nguyen, D; Dierckens, K; Boon, N; Lacoere, T; Kerckhof, FM; De Vrieze, J; Vadstein, O; Bossier, P | 2020 | Gut Microbiota of Migrating Wild Rabbit Fish (Siganus guttatus) Larvae Have Low Spatial and Temporal Variability | **Prokaryote** | 16S | Fish | <http://dx.doi.org/10.1007/s00248-019-01436-1> |
| 70 | | Renaud, V; Houde, VP; Pilon, G; Varin, TV; Roblet, C; Marette, A; Boutin, Y; Bazinet, L | 2021 | The Concentration of Organic Acids in Cranberry Juice Modulates the Gut Microbiota in Mice | **Prokaryote** | 16S | Mice | <http://dx.doi.org/10.3390/ijms222111537> |
| 71 | | Zhao, Z; Ning, JW; Bao, XQ; Shang, MY; Ma, JW; Li, G; Zhang, D | 2021 | Fecal microbiota transplantation protects rotenone-induced Parkinson's disease mice via suppressing inflammation mediated by the lipopolysaccharide-TLR4 signaling pathway through the microbiota-gut-brain axis | **Prokaryote** | 16S | Mice | <http://dx.doi.org/10.1186/s40168-021-01107-9> |
| 72 | | Constante, M; De Palma, G; Lu, J; Jury, J; Rondeau, L; Caminero, A; Collins, SM; Verdu, EF; Bercik, P | 2021 | Saccharomyces boulardiiCNCM I-745 modulates the microbiota-gut-brain axis in a humanized mouse model of Irritable Bowel Syndrome | **Prokaryote** | 16S | Mice | <http://dx.doi.org/10.1111/nmo.13985> |
| 73 | | Zheng, JH; Li, H; Zhang, P; Yue, SJ; Zhai, BT; Zou, JB; Cheng, JX; Zhao, CB; Guo, DY; Wang, J | 2022 | Paeonol Ameliorates Ulcerative Colitis in Mice by Modulating the Gut Microbiota and Metabolites | **Prokaryote** | 16S | Mice | <http://dx.doi.org/10.3390/metabo12100956> |
| 74 | | Nishiyama, K; Aono, K; Fujimoto, Y; Kuwamura, M; Okada, T; Tokumoto, H; Izawa, T; Okano, R; Nakajima, H; Takeuchi, T; Azuma, YT | 2019 | Chronic kidney disease after 5/6 nephrectomy disturbs the intestinal microbiota and alters intestinal motility | **Prokaryote** | 16S | Mice | <http://dx.doi.org/10.1002/jcp.27408> |
| 75 | | He, CX; Wu, HD; Lv, YN; You, HN; Zha, LQ; Li, Q; Huang, YN; Tian, JH; Chen, QC; Shen, YW; Xiong, SY; Xue, FG | 2021 | Gastrointestinal Development and Microbiota Responses of Geese to Honeycomb Flavonoids Supplementation | **Prokaryote** | 16S | Birds | <http://dx.doi.org/10.3389/fvets.2021.739237> |
| 76 | | Jiang, Y; Liu, ZX; Liu, XZ; Xu, YJ; Shi, B; Wang, B | 2019 | Structural characteristics and succession of intestinal microbiota for Paralichthys olivaceus during the early life stage | **Prokaryote** | 16S | Fish | <http://dx.doi.org/10.1111/are.13924> |
| 77 | | Goya-Jorge, E; Gonza, I; Bondue, P; Douny, C; Taminiau, B; Daube, G; Scippo, ML; Delcenserie, V | 2022 | Human Adult Microbiota in a Static Colon Model: AhR Transcriptional Activity at the Crossroads of Host-Microbe Interaction | **Prokaryote** | 16S | Human | <http://dx.doi.org/10.3390/foods11131946> |
| 78 | | Chleilat, F; Klancic, T; Ma, K; Schick, A; Nettleton, JE; Reimer, RA | 2020 | Human Milk Oligosaccharide Supplementation Affects Intestinal Barrier Function and Microbial Composition in the Gastrointestinal Tract of Young Sprague Dawley Rats | **Prokaryote** | 16S | Rats | <http://dx.doi.org/10.3390/nu12051532> |
| 79 | | Palacios-Gonzalez, B; Meraz-Cruz, N; Valdez-Palomares, F; Nambo-Venegas, R | 2019 | Equibiotic-GI Consumption Improves Intestinal Microbiota in Subjects with Functional Dyspepsia | **Prokaryote** | 16S | Human | <http://dx.doi.org/10.2174/1574885514666190212114412> |
| 80 | | Karl, JP; Berryman, CE; Young, AJ; Radcliffe, PN; Branck, TA; Pantoja-Feliciano, IG; Rood, JC; Pasiakos, SM | 2018 | Associations between the gut microbiota and host responses to high altitude | **Prokaryote** | 16S | Human | <http://dx.doi.org/10.1152/ajpgi.00253.2018> |
| 81 | | Kim, JW; Jeong, Y; Park, SJ; Jin, H; Lee, J; Ju, JH; Ji, GE; Park, SH | 2021 | Influence of proton pump inhibitor or rebamipide use on gut microbiota of rheumatoid arthritis patients | **Prokaryote** | 16S | Human | <http://dx.doi.org/10.1093/rheumatology/keaa316> |
| 82 | | Zhang, Z; Lim, X; Huang, Y; Zhou, JW; Yang, SS; Wei, XY; Lai, WJ; Zhang, X; Fu, CM | 2021 | An Integrated Gut Microbiota and Network Pharmacology Study on Fuzi-Lizhong Pill for Treating Diarrhea-Predominant Irritable Bowel Syndrome | **Prokaryote** | 16S | Rats | <http://dx.doi.org/10.3389/fphar.2021.746923> |
| 83 | | Xu, FH; Li, N; Wang, C; Xing, HY; Chen, DF; Wei, YL | 2021 | Clinical efficacy of fecal microbiota transplantation for patients with small intestinal bacterial overgrowth: a randomized, placebo-controlled clinic study | **Prokaryote** | 16S | Human | <http://dx.doi.org/10.1186/s12876-021-01630-x> |
| 84 | | Larsen, IS; Jensen, BAH; Bonazzi, E; Choi, BSY; Kristensen, NN; Schmidt, EGW; Suenderhauf, A; Morin, L; Olsen, PB; Hansen, LBS; Schroder, T; Sina, C; Chassaing, B; Marette, A | 2021 | Fungal lysozyme leverages the gut microbiota to curb DSS-induced colitis | **Prokaryote** | 16S | Mice | <http://dx.doi.org/10.1080/19490976.2021.1988836> |
| 85 | | Li, WH; Liu, JM; Tan, H; Yang, CH; Ren, L; Liu, QF; Wang, S; Hu, FZ; Xiao, J; Zhao, RR; Tao, M; Zhang, C; Qin, QB; Liu, SJ | 2018 | Genetic Effects on the Gut Microbiota Assemblages of Hybrid Fish From Parents With Different Feeding Habits | **Prokaryote** | 16S | Fish | <http://dx.doi.org/10.3389/fmicb.2018.02972> |
| 86 | | Pang, B; Jin, H; Liao, N; Li, JJ; Jiang, CM; Shi, JL | 2021 | Vitamin A supplementation ameliorates ulcerative colitis in gut microbiota-dependent manner | **Prokaryote** | 16S | Mice | <http://dx.doi.org/10.1016/j.foodres.2021.110568> |
| 87 | | Chen, GW; Wang, G; Zhu, CJ; Jiang, XW; Sun, JX; Tian, LM; Bai, WB | 2019 | Effects of cyanidin-3-O-glucoside on 3-chloro-1,2-propanediol induced intestinal microbiota dysbiosis in rats | **Prokaryote** | 16S | Rats | <http://dx.doi.org/10.1016/j.fct.2019.110767> |
| 88 | | Zhou, BL; Xia, XY; Wang, PQ; Chen, S; Yu, CH; Huang, R; Zhang, R; Wang, YT; Lu, L; Yuan, FJ; Tian, YM; Fan, YZ; Zhang, XY; Shu, Y; Zhang, SY; Bai, D; Wu, L; Xu, H; Yan, L | 2018 | Induction and Amelioration of Methotrexate-Induced Gastrointestinal Toxicity are Related to Immune Response and Gut Microbiota | **Prokaryote** | 16S | Mice | <http://dx.doi.org/10.1016/j.ebiom.2018.06.029> |
| 89 | | Zhuang, M; Shang, WT; Ma, QC; Strappe, P; Zhou, ZK | 2019 | Abundance of Probiotics and Butyrate-Production Microbiome Manages Constipation via Short-Chain Fatty Acids Production and Hormones Secretion | **Prokaryote** | 16S | Humans-Rats | <http://dx.doi.org/10.1002/mnfr.201801187> |
| 90 | | Alatawi, H; Mosli, M; Saadah, OI; Annese, V; Al-Hindi, R; Alatawy, M; Al-Amrah, H; Alshehri, D; Bahieldin, A; Edris, S | 2022 | Attributes of intestinal microbiota composition and their correlation with clinical primary non-response to anti-TNF-alpha agents in inflammatory bowel disease patients | **Prokaryote** | 16S | Human | <http://dx.doi.org/10.17305/bjbms.2021.6436> |
| 91 | | He, Y; Zhu, LL; Chen, JL; Tang, X; Pan, ML; Yuan, WW; Wang, HC | 2022 | Efficacy of Probiotic Compounds in Relieving Constipation and Their Colonization in Gut Microbiota | **Prokaryote** | 16S | Mice | <http://dx.doi.org/10.3390/molecules27030666> |
| 92 | | Goloshchapov, OV; Olekhnovich, EI; Sidorenko, SV; Moiseev, IS; Kucher, MA; Fedorov, DE; Pavlenko, AV; Manolov, AI; Gostev, VV; Veselovsky, VA; Klimina, KM; Kostryukova, ES; Bakin, EA; Shvetcov, AN; Gumbatova, ED; Klementeva, RV; Shcherbakov, AA; Gorchakova, MV; Egozcue, JJ; Pawlowsky-Glahn, V; Suvorova, MA; Chukhlovin, AB; Govorun, VM; Ilina, EN; Afanasyev, BV | 2019 | Long-term impact of fecal transplantation in healthy volunteers | **Prokaryote** | 16S | Human | <http://dx.doi.org/10.1186/s12866-019-1689-y> |
| 93 | | Pensec, C; Gillaizeau, F; Guenot, D; Bessard, A; Carton, T; Leuillet, S; Campone, M; Neunlist, M; Blottiere, HM; Le Vacon, F | 2020 | Impact of pemetrexed chemotherapy on the gut microbiota and intestinal inflammation of patient-lung-derived tumor xenograft (PDX) mouse models | **Prokaryote** | 16S | Mice | <http://dx.doi.org/10.1038/s41598-020-65792-6> |
| 94 | | Horne, R; St Pierre, J; Odeh, S; Surette, M; Foster, JA | 2019 | Microbe and host interaction in gastrointestinal homeostasis | **Prokaryote** | 16S | Mice | <http://dx.doi.org/10.1007/s00213-019-05218-y> |
| 95 | | Mueller, KD; Zhang, H; Serrano, CR; Billmyre, RB; Huh, EY; Wiemann, P; Keller, NP; Wang, YF; Heitman, J; Lee, SC | 2019 | Gastrointestinal microbiota alteration induced by Mucor circinelloides in a murine model | **Eukaryote + Prokaryote** | ITS + 16S | Mice | <http://dx.doi.org/10.1007/s12275-019-8682-x> |
| 96 | | Mennini, M; Reddel, S; Del Chierico, F; Gardini, S; Quagliariello, A; Vernocchi, P; Valluzzi, RL; Fierro, V; Riccardi, C; Napolitano, T; Fiocchi, AG; Putignani, L | 2021 | Gut Microbiota Profile in Children with IgE-Mediated Cow's Milk Allergy and Cow's Milk Sensitization and Probiotic Intestinal Persistence Evaluation | **Eukaryote + Prokaryote** | ITS + 16S | Human | <http://dx.doi.org/10.3390/ijms22041649> |
| 97 | | Dong, SJ; Zhu, M; Wang, K; Zhao, XY; Hu, LL; Jing, WH; Lu, HT; Wang, SC | 2021 | Dihydromyricetin improves DSS-induced colitis in mice via modulation of fecal-bacteria-related bile acid metabolism | **Prokaryote** | 16S | Mice | <http://dx.doi.org/10.1016/j.phrs.2021.105767> |
| 98 | | Jokela, R; Korpela, K; Jian, C; Dikareva, E; Nikkonen, A; Saisto, T; Skogberg, K; de Vos, WM; Kolho, KL; Salonen, A | 2022 | Quantitative insights into effects of intrapartum antibiotics and birth mode on infant gut microbiota in relation to well-being during the first year of life | **Prokaryote** | 16S | Human | <http://dx.doi.org/10.1080/19490976.2022.2095775> |
| 99 | | Zhang, Y; Wen, B; Meng, LJ; Gao, JZ; Chen, ZZ | 2021 | Dynamic changes of gut microbiota of discus fish (Symphysodon haraldi) at different feeding stages | **Prokaryote** | 16S | Fish | <http://dx.doi.org/10.1016/j.aquaculture.2020.735912> |
| 100 | | Cotozzolo, E; Cremonesi, P; Curone, G; Menchetti, L; Riva, F; Biscarini, F; Marongiu, ML; Castrica, M; Castiglioni, B; Miraglia, D; Luridiana, S; Brecchia, G | 2021 | Characterization of Bacterial Microbiota Composition along the Gastrointestinal Tract in Rabbits | **Prokaryote** | 16S | Rabbits | <https://www.mdpi.com/2076-2615/11/1/31> |
| 101 | | Cheng, J; Li, WM; Wang, Y; Cao, Q; Ni, Y; Zhang, WY; Guo, JB; Chen, BL; Zang, YN; Zhu, Y | 2022 | Electroacupuncture modulates the intestinal microecology to improve intestinal motility in spinal cord injury rats | **Prokaryote** | 16S | Rats | <http://dx.doi.org/10.1111/1751-7915.13968> |
| 102 | | Lun, HZ; Yang, WH; Zhao, SP; Jiang, MJ; Xu, MJ; Liu, FF; Wang, YS | 2019 | Altered gut microbiota and microbial biomarkers associated with chronic kidney disease | **Prokaryote** | 16S | Human | <http://dx.doi.org/10.1002/mbo3.678> |
| 103 | | Hetemaki, I; Jian, C; Laakso, S; Makitie, O; Pajari, AM; de Vos, WM; Arstila, TP; Salonen, A | 2021 | Fecal Bacteria Implicated in Biofilm Production Are Enriched and Associate to Gastrointestinal Symptoms in Patients With APECED - A Pilot Study | **Eukaryote + Prokaryote** | ITS + 16S | Human | <http://dx.doi.org/10.3389/fimmu.2021.668219> |
| 104 | | Feng, WW; Zhang, J; Jakovlic, I; Xiong, F; Wu, SG; Zou, H; Li, WX; Li, M; Wang, GT | 2019 | Gut segments outweigh the diet in shaping the intestinal microbiota composition in grass carp Ctenopharyngodon idellus | **Prokaryote** | 16S | Fish | <http://dx.doi.org/10.1186/s13568-019-0770-0> |
| 105 | | Xu, CAH; Liu, JH; Gao, JW; Wu, XY; Cui, CB; Wei, HK; Peng, J; Zheng, R | 2021 | The Effect of Functional Fiber on Microbiota Composition in Different Intestinal Segments of Obese Mice | **Prokaryote** | 16S | Mice | <http://dx.doi.org/10.3390/ijms22126525> |
| 106 | | Jin, XK; Chen, ZW; Shi, Y; Gui, JF; Zhao, Z | 2022 | Response of gut microbiota to feed-borne bacteria depends on fish growth rate: a snapshot survey of farmed juvenile Takifugu obscurus | **Prokaryote** | 16S | Fish | <http://dx.doi.org/10.1111/1751-7915.13741> |
| 107 | | Chang, CW; Lee, HC; Li, LH; Chiau, JSC; Wang, TE; Chuang, WH; Chen, MJ; Wang, HY; Shih, SC; Liu, CY; Tsai, TH; Chen, YJ | 2020 | Fecal Microbiota Transplantation Prevents Intestinal Injury, Upregulation of Toll-Like Receptors, and 5-Fluorouracil/Oxaliplatin-Induced Toxicity in Colorectal Cancer | **Prokaryote** | 16S | Mice | <http://dx.doi.org/10.3390/ijms21020386> |
| 108 | | Zhang, YY; Gan, Y; Wang, J; Feng, ZX; Zhong, ZX; Bao, HD; Xiong, QY; Wang, R | 2022 | Dysbiosis of Gut Microbiota and Intestinal Barrier Dysfunction in Pigs with Pulmonary Inflammation Induced by Mycoplasma hyorhinis Infection | **Prokaryote** | 16S | Pigs | <http://dx.doi.org/10.1128/msystems.00282-22> |
| 109 | | Kers, JG; Velkers, FC; Fischer, EAJ; Stegeman, JA; Smidt, H; Hermes, GDA | 2022 | Conserved developmental trajectories of the cecal microbiota of broiler chickens in a field study | **Prokaryote** | 16S | Birds | <http://dx.doi.org/10.1093/femsec/fiac090> |
| 110 | | Li, X; Wang, L; Ma, SJ; Lin, SH; Wang, CY; Wang, HY | 2022 | Combination of Oxalobacter Formigenes and Veillonella Parvula in Gastrointestinal Microbiota Related to Bile-Acid Metabolism as a Biomarker for Hypertensive Nephropathy | **Prokaryote** | 16S | Human | <http://dx.doi.org/10.1155/2022/5999530> |
| 111 | | Fung, TC; Vuong, HE; Luna, CDG; Pronovost, GN; Aleksandrova, AA; Riley, NG; Vavilina, A; McGinn, J; Rendon, T; Forrest, LR; Hsiao, EY | 2019 | Intestinal serotonin and fluoxetine exposure modulate bacterial colonization in the gut | **Prokaryote** | 16S | Mice | <http://dx.doi.org/10.1038/s41564-019-0540-4> |
| 112 | | Xu, MM; Wang, L; Guo, Y; Zhang, W; Chen, Y; Li, Y | 2021 | Positive Effect of Electroacupuncture Treatment on Gut Motility in Constipated Mice Is Related to Rebalancing the Gut Microbiota | **Prokaryote** | 16S | Mice | <http://dx.doi.org/10.1155/2021/6652017> |
| 113 | | Sun, ZM; Li, JX; Dai, Y; Wang, WT; Shi, R; Wang, ZB; Ding, PH; Lu, QQ; Jiang, H; Pei, WJ; Zhao, XJ; Guo, Y; Liu, JL; Tan, X; Mao, TY | 2020 | Indigo Naturalis Alleviates Dextran Sulfate Sodium-Induced Colitis in Rats via Altering Gut Microbiota | **Prokaryote** | 16S | Mice | <http://dx.doi.org/10.3389/fmicb.2020.00731> |
| 114 | | Sun, CA; Chen, L; Yang, H; Sun, HJ; Xie, Z; Zhao, B; Jiang, XM; Qin, B; Shen, Z | 2021 | Involvement of Gut Microbiota in the Development of Psoriasis Vulgaris | **Prokaryote** | 16S | Human | <http://dx.doi.org/10.3389/fnut.2021.761978> |
| 115 | | Lei, ZL; Chen, L; Hu, Q; Yang, YH; Tong, FX; Li, KY; Lin, T; Nie, Y; Rong, HD; Yu, SP; Song, Q; Guo, J | 2022 | Ginsenoside Rb1 improves intestinal aging via regulating the expression of sirtuins in the intestinal epithelium and modulating the gut microbiota of mice | **Prokaryote** | 16S | Mice | <http://dx.doi.org/10.3389/fphar.2022.991597> |
| 116 | | Zhao, RX; Symonds, JE; Walker, SP; Steiner, K; Carter, CG; Bowman, JP; Nowak, BF | 2020 | Salinity and fish age affect the gut microbiota of farmed Chinook salmon (Oncorhynchus tshawytscha) | **Prokaryote** | 16S | Fish | <http://dx.doi.org/10.1016/j.aquaculture.2020.735539> |
| 117 | | Mortensen, MS; Jensen, BH; Williams, J; Brejnrod, AD; Andersen, LO; Roser, D; Andreassen, BU; Petersen, AM; Stensvold, CR; Sorensen, SJ; Krogfelt, KA | 2018 | Stability and resilience of the intestinal microbiota in children in daycare - a 12month cohort study | **Prokaryote** | 16S | Human | <http://dx.doi.org/10.1186/s12866-018-1367-5> |
| 118 | | Landsiedel, R; Hahn, D; Ossig, R; Ritz, S; Sauer, L; Buesen, R; Rehm, S; Wohlleben, W; Groeters, S; Strauss, V; Sperber, S; Wami, H; Dobrindt, U; Prior, K; Harmsen, D; van Ravenzwaay, B; Schnekenburger, J | 2022 | Gut microbiome and plasma metabolome changes in rats after oral gavage of nanoparticles: sensitive indicators of possible adverse health effects | **Prokaryote** | 16S | Rats | <http://dx.doi.org/10.1186/s12989-022-00459-w> |
| 119 | | Poteres, E; Hubert, N; Poludasu, S; Brigando, G; Moore, J; Keeler, K; Isabelli, A; Ibay, ICV; Alt, L; Pytynia, M; Ciancio, M; Martinez-Guryn, K | 2020 | Selective Regional Alteration of the Gut Microbiota by Diet and Antibiotics | **Prokaryote** | 16S | Mice | <http://dx.doi.org/10.3389/fphys.2020.00797> |
| 120 | | Mizutani, T; Ishizaka, A; Koga, M; Ikeuchi, K; Saito, M; Adachi, E; Yamayoshi, S; Iwatsuki-Horimoto, K; Yasuhara, A; Kiyono, H; Matano, T; Suzuki, Y; Tsutsumi, T; Kawaoka, Y; Yotsuyanagi, H | 2022 | Correlation Analysis between Gut Microbiota Alterations and the Cytokine Response in Patients with Coronavirus Disease during Hospitalization | **Prokaryote** | 16S | Human | <http://dx.doi.org/10.1128/spectrum.01689-21> |
| 121 | | Lai, F; Jiang, R; Xie, WJ; Liu, XR; Tang, Y; Xiao, H; Gao, JY; Jia, Y; Bai, QH | 2018 | Intestinal Pathology and Gut Microbiota Alterations in a Methyl-4-phenyl-1,2,3,6-tetrahydropyridine (MPTP) Mouse Model of Parkinson's Disease | **Prokaryote** | 16S | Mice | <http://dx.doi.org/10.1007/s11064-018-2620-x> |
| 122 | | Wu, DT; Fu, Y; Guo, H; Yuan, Q; Nie, XR; Wang, SP; Gan, RY | 2021 | In vitro simulated digestion and fecal fermentation of polysaccharides from loquat leaves: Dynamic changes in physicochemical properties and impacts on human gut microbiota | **Prokaryote** | 16S | Culture (in vitro) | <http://dx.doi.org/10.1016/j.ijbiomac.2020.11.130> |
| 123 | | Seekatz, AM; Schnizlein, MK; Koenigsknecht, MJ; Baker, JR; Hasler, WL; Bleske, BE; Young, VB; Sun, DX | 2019 | Spatial and Temporal Analysis of the Stomach and Small-Intestinal Microbiota in Fasted Healthy Humans | **Prokaryote** | 16S | Human | <http://dx.doi.org/10.1128/mSphere.00126-19> |
| 124 | | Han, C; Guo, NN; Bu, Y; Peng, YH; Li, XT; Ma, XH; Yang, MY; Jia, XN; Zhang, J; Liu, XW; Yu, KJ; Wang, CS | 2021 | Intestinal microbiota and antibiotic-associated acute gastrointestinal injury in sepsis mice | **Prokaryote** | 16S | Mice | <https://www.ncbi.nlm.nih.gov/pmc/articles/PMC8064167/> |
| 125 | | Karl, JP; Armstrong, NJ; Player, RA; Rood, JC; Soares, JW; McClung, HL | 2022 | The Fecal Metabolome Links Diet Composition, Foacidic positive ion conditions, chromatographicallyod Processing, and the Gut Microbiota to Gastrointestinal Health in a Randomized Trial of Adults Consuming a Processed Diet | **Prokaryote** | 16S | Human | <http://dx.doi.org/10.1093/jn/nxac161> |
| 126 | | Zeng, A; Tan, K; Gong, P; Lei, P; Guo, ZH; Wang, SP; Gao, SF; Zhou, YH; Shu, Y; Zhou, XL; Miao, D; Zeng, FJ; Liu, HZ | 2020 | Correlation of microbiota in the gut of fish species and water | **Prokaryote** | 16S | Fish/water | <http://dx.doi.org/10.1007/s13205-020-02461-5> |
| 127 | | Li, WQ; Chen, MX; Feng, X; Song, M; Shao, ML; Yang, YF; Zhang, LW; Liu, Q; Lv, LX; Su, X | 2021 | Maternal immune activation alters adult behavior, intestinal integrity, gut microbiota and the gut inflammation | **Prokaryote** | 16S | Rats | <http://dx.doi.org/10.1002/brb3.2133> |
| 128 | | Choudhury, R; Middelkoop, A; de Souza, JG; van Veen, LA; Gerrits, WJJ; Kemp, B; Bolhuis, JE; Kleerebezem, M | 2021 | Impact of early-life feeding on local intestinal microbiota and digestive system development in piglets | **Prokaryote** | 16S | Pig | <http://dx.doi.org/10.1038/s41598-021-83756-2> |
| 129 | | Muller, PA; Schneeberger, M; Matheis, F; Wang, PTQ; Kerner, Z; Ilanges, A; Pellegrino, K; del Marmol, J; Castro, TBR; Furuichi, M; Perkins, M; Han, WF; Rao, A; Picard, AJ; Cross, JR; Honda, K; de Araujo, I; Mucida, D | 2020 | Microbiota modulate sympathetic neurons via a gut-brain circuit | **Prokaryote** | 16S | Mice | <http://dx.doi.org/10.1038/s41586-020-2474-7> |
| 130 | | van Best, N; Rolle-Kampczyk, U; Schaap, FG; Basic, M; Damink, SWMO; Bleich, A; Savelkoul, PHM; von Bergen, M; Penders, J; Hornef, MW | 2020 | Bile acids drive the newborn's gut microbiota maturation | **Prokaryote** | 16S | Mice | <http://dx.doi.org/10.1038/s41467-020-17183-8> |
| 131 | | Bai, YJ; Zhou, Y; Zhang, RF; Chen, YX; Wang, FZ; Zhang, MW | 2023 | Gut microbial fermentation promotes the intestinal anti-inflammatory activity of Chinese yam polysaccharides | **Prokaryote** | 16S | in vitro | <http://dx.doi.org/10.1016/j.foodchem.2022.134003> |
| 132 | | Bellocchi, C; Fernandez-Ochoa, A; Montanelli, G; Vigone, B; Santaniello, A; Milani, C; Quirantes-Pine, R; Borras-Linares, I; Ventura, M; Segura-Carrettero, A; Alarcon-Riquelme, ME; Beretta, L | 2018 | Microbial and metabolic multi-omic correlations in systemic sclerosis patients | **Prokaryote** | 16S | Human | <http://dx.doi.org/10.1111/nyas.13736> |
| 133 | | Okamoto, K; Watanabe, T; Komeda, Y; Okamoto, A; Minaga, K; Kamata, K; Yamao, K; Takenaka, M; Hagiwara, S; Sakurai, T; Tanaka, T; Sakamoto, H; Fujimoto, K; Nishida, N; Kudo, M | 2018 | Dysbiosis-Associated Polyposis of the Colon-Cap Polyposis | **Prokaryote** | 16S | Human | <http://dx.doi.org/10.3389/fimmu.2018.00918> |
| 134 | | Peng, SY; Ling, XW; Rui, WJ; Jin, XB; Chu, FJ | 2022 | LMWP (S3-3) from the Larvae of Musca domestica Alleviate D-IBS by Adjusting the Gut Microbiota | **Prokaryote** | 16S | Mice | <http://dx.doi.org/10.3390/molecules27144517> |
| 135 | | Li, Y; Xiao, HW; Dong, JL; Luo, D; Wang, HC; Zhang, SQ; Zhu, T; Zhu, CC; Cui, M; Fan, SJ | 2020 | Gut Microbiota Metabolite Fights Against Dietary Polysorbate 80-Aggravated Radiation Enteritis | **Prokaryote** | 16S | Mice | <http://dx.doi.org/10.3389/fmicb.2020.01450> |
| 136 | | Wilkinson, N; Hughes, RJ; Bajagai, YS; Aspden, WJ; Van, TTH; Moore, RJ; Stanley, D | 2020 | Reduced environmental bacterial load during early development and gut colonisation has detrimental health consequences in Japanese quail | **Prokaryote** | 16S | Birds | <http://dx.doi.org/10.1016/j.heliyon.2020.e03213> |
| 137 | | Chen, H; Ma, XM; Liu, YY; Ma, LL; Chen, ZY; Lin, XL; Si, L; Ma, XY; Chen, XH | 2019 | Gut Microbiota Interventions With Clostridium butyricum and Norfloxacin Modulate Immune Response in Experimental Autoimmune Encephalomyelitis Mice | **Prokaryote** | 16S | Mice | <http://dx.doi.org/10.3389/fimmu.2019.01662> |
| 138 | | Li, YY; Long, SQ; Liu, QC; Ma, H; Li, JN; Wei, XQ; Yuan, JL; Li, M; Hou, BB | 2020 | Gut microbiota is involved in the alleviation of loperamide-induced constipation by honey supplementation in mice | **Prokaryote** | 16S | Mice | <http://dx.doi.org/10.1002/fsn3.1736> |
| 139 | | Moreira-Rosario, A; Marques, C; Pinheiro, H; Norberto, S; Sintra, D; Teixeira, JA; Calhau, C; Azevedo, LF | 2020 | Daily intake of wheat germ-enriched bread may promote a healthy gut bacterial microbiota: a randomised controlled trial | **Prokaryote** | 16S | Human | <http://dx.doi.org/10.1007/s00394-019-02045-x> |
| 140 | | Zheng, R; Wang, PJ; Cao, BP; Wu, MY; Li, XY; Wang, HY; Chai, LH | 2021 | Intestinal response characteristic and potential microbial dysbiosis in digestive tract of Bufo gargarizans after exposure to cadmium and lead, alone or combined | **Prokaryote** | 16S | Amphibian | <http://dx.doi.org/10.1016/j.chemosphere.2020.129511> |
| 141 | | Sparagon, WJ; Gentry, EC; Minich, JJ; Vollbrecht, L; Laurens, LML; Allen, EE; Sims, NA; Dorrestein, PC; Kelly, LW; Nelson, CE | 2022 | Fine scale transitions of the microbiota and metabolome along the gastrointestinal tract of herbivorous fishes | **Prokaryote** | 16S | fish | <http://dx.doi.org/10.1186/s42523-022-00182-z> |
| 142 | | Liu, C; Huang, SM; Wu, ZH; Li, TT; Li, N; Zhang, B; Han, DD; Wang, SL; Zhao, JC; Wang, JJ | 2021 | Cohousing-mediated microbiota transfer from milk bioactive components-dosed mice ameliorate colitis by remodeling colonic mucus barrier and lamina propria macrophages | **Prokaryote** | 16S | Mice | <http://dx.doi.org/10.1080/19490976.2021.1903826> |
| 143 | | Hu, YOO; Hugerth, LW; Bengtsson, C; Alisjahbana, A; Seifert, M; Kamal, A; Sjoling, A; Midtvedt, T; Norin, E; Du, J; Engstrand, L | 2018 | Bacteriophages Synergize with the Gut Microbial Community To Combat Salmonella | **Prokaryote** | 16S | in vitro | <http://dx.doi.org/10.1128/mSystems.00119-18> |
| 144 | | Nolorbe-Payahua, CD; de Freitas, AS; Roesch, LFW; Zanette, J | 2020 | Environmental contamination alters the intestinal microbial community of the livebearer killifish Phalloceros caudimaculatus | **Prokaryote** | 16S | Fish | <http://dx.doi.org/10.1016/j.heliyon.2020.e04190> |
| 145 | | Zhang, Q; Zhong, D; Sun, R; Zhang, Y; Pegg, RB; Zhong, G | 2021 | Prevention of loperamide induced constipation in mice by KGM and the mechanisms of different gastrointestinal tract microbiota regulation | **Prokaryote** | 16S | Mice | <http://dx.doi.org/10.1016/j.carbpol.2020.117418> |
| 146 | | Afrin, T; Murase, K; Kounosu, A; Hunt, VL; Bligh, M; Maeda, Y; Hino, A; Maruyama, H; Tsai, IJ; Kikuchi, T | 2019 | Sequential Changes in the Host Gut Microbiota During Infection With the Intestinal Parasitic Nematode Strongyloides venezuelensis | **Prokaryote** | 16S | Mice | <http://dx.doi.org/10.3389/fcimb.2019.00217> |
| 147 | | Ishida, T; Jobu, K; Kawada, K; Morisawa, S; Kawazoe, T; Shiraishi, H; Fujita, H; Nishimura, S; Kanno, H; Nishiyama, M; Ogawa, K; Morita, Y; Hanazaki, K; Miyamura, M | 2022 | Impact of Gut Microbiota on the Pharmacokinetics of Glycyrrhizic Acid in Yokukansan, a Kampo Medicine | **Prokaryote** | 16S | Mice | <https://www.jstage.jst.go.jp/article/bpb/45/1/45_b21-00658/_article/-char/ja/> |
| 148 | | Edogawa, S; Peters, SA; Jenkins, GD; Gurunathan, SV; Sundt, WJ; Johnson, S; Lennon, RJ; Dyer, RB; Camilleri, M; Kashyap, PC; Farrugia, G; Chen, J; Singh, RJ; Grover, M | 2018 | Sex differences in NSAID-induced perturbation of human intestinal barrier function and microbiota | **Prokaryote** | 16S | Human | <http://dx.doi.org/10.1096/fj.201800560R> |
| 149 | | Salgaco, MK; Perina, NP; Tome, TM; Mosquera, EMB; Lazarini, T; Sartoratto, A; Sivieri, K | 2021 | Probiotic infant cereal improves children's gut microbiota: Insights using the Simulator of Human Intestinal Microbial Ecosystem (SHIME (R)) | **Prokaryote** | 16S | Human | <http://dx.doi.org/10.1016/j.foodres.2021.110292> |
| 150 | | Jakobsen, LMA; Sundekilde, UK; Andersen, HJ; Kot, W; Mejia, JLC; Nielsen, DS; Hansen, AK; Bertram, HC | 2021 | Administration of Bovine Milk Oligosaccharide to Weaning Gnotobiotic Mice Inoculated with a Simplified Infant Type Microbiota | **Prokaryote** | 16S | Mice | <http://dx.doi.org/10.3390/microorganisms9051003> |
| 151 | | Heimesaat, MM; Genger, C; Biesemeier, N; Klove, S; Weschka, D; Mousavi, S; Bereswill, S | 2020 | Inflammatory Immune Responses and Gut Microbiota Changes Following Campylobacter coli Infection of IL-10(-/-)Mice with Chronic Colitis | **Prokaryote** | 16S | Mice | <http://dx.doi.org/10.3390/pathogens9070560> |
| 152 | | Chen, L; Li, SX; Xiao, Q; Lin, Y; Li, XX; Qu, YF; Wu, GG; Li, H | 2021 | Composition and diversity of gut microbiota in Pomacea canaliculata in sexes and between developmental stages | **Prokaryote** | 16S | snails | <http://dx.doi.org/10.1186/s12866-021-02259-2> |
| 153 | | Gao, XY; Hu, YF; Tao, YF; Liu, SF; Chen, HW; Li, JY; Zhao, Y; Sheng, J; Tian, Y; Fan, YH | 2022 | Cymbopogon citratus (DC.) Stapf aqueous extract ameliorates loperamide-induced constipation in mice by promoting gastrointestinal motility and regulating the gut microbiota | **Prokaryote** | 16S | Human | <http://dx.doi.org/10.3389/fmicb.2022.1017804> |
| 154 | | Chen, XL; Yi, HD; Liu, S; Zhang, Y; Su, YQ; Liu, XG; Bi, S; Lai, H; Zeng, ZY; Li, GF | 2021 | Probiotics Improve Eating Disorders in Mandarin Fish (Siniperca chuatsi) Induced by a Pellet Feed Diet via Stimulating Immunity and Regulating Gut Microbiota | **Prokaryote** | 16S | Fish | <http://dx.doi.org/10.3390/microorganisms9061288> |
| 155 | | Garibay-Valdez, E; Cicala, F; Martinez-Porchas, M; Gomez-Reyes, R; Vargas-Albores, F; Gollas-Galvan, T; Martinez-Cordova, LR; Calderon, K | 2021 | Longitudinal variations in the gastrointestinal microbiome of the white shrimp, Litopenaeus vannamei | **Prokaryote** | 16S | Shrimp | <http://dx.doi.org/10.7717/peerj.11827> |
| 156 | | Kuang, TX; He, AY; Lin, YF; Huang, XD; Liu, L; Zhou, L | 2020 | Comparative analysis of microbial communities associated with the gill, gut, and habitat of two filter-feeding fish | **Prokaryote** | 16S | Fish | <http://dx.doi.org/10.1016/j.aqrep.2020.100501> |
| 157 | | Nakajima, H; Takewaki, F; Hashimoto, Y; Kajiyama, S; Majima, S; Okada, H; Senmaru, T; Ushigome, E; Nakanishi, N; Hamaguchi, M; Yamazaki, M; Tanaka, Y; Oikawa, Y; Nakajima, S; Ohno, H; Fukui, M | 2020 | The Effects of Metformin on the Gut Microbiota of Patients with Type 2 Diabetes: A Two-Center, Quasi-Experimental Study | **Prokaryote** | 16S | Human | <http://dx.doi.org/10.3390/life10090195> |
| 158 | | Gresse, R; Durand, FC; Duniere, L; Blanquet-Diot, S; Forano, E | 2019 | Microbiota Composition and Functional Profiling Throughout the Gastrointestinal Tract of Commercial Weaning Piglets | **Prokaryote** | 16S | Pigs | <http://dx.doi.org/10.3390/microorganisms7090343> |
| 159 | | Hou, YF; Dong, L; Lu, XL; Shi, HT; Xu, B; Zhong, WT; Ma, L; Wang, SH; Yang, CF; He, XY; Zhao, YD; Wang, SH | 2022 | Distinctions Between Fecal and Intestinal Mucosal Microbiota in Subgroups of Irritable Bowel Syndrome | **Prokaryote** | 16S | Human | <http://dx.doi.org/10.1007/s10620-022-07588-4> |
| 160 | | Chen, CM; Chou, HC; Yang, YCSH | 2021 | Maternal Antibiotic Treatment Disrupts the Intestinal Microbiota and Intestinal Development in Neonatal Mice | **Prokaryote** | 16S | Mice | <http://dx.doi.org/10.3389/fmicb.2021.684233> |
| 161 | | Zhang, HH; Liu, J; Lv, YJ; Jiang, YL; Pan, JX; Zhu, YJ; Huang, MG; Zhang, SK | 2020 | Changes in Intestinal Microbiota of Type 2 Diabetes in Mice in Response to Dietary Supplementation With Instant Tea or Matcha | **Prokaryote** | 16S | Mice | <http://dx.doi.org/10.1016/j.jcjd.2019.04.021> |
| 162 | | Kulas, J; Mirkov, I; Tucovic, D; Zolotarevski, L; Glamoclija, J; Veljovic, K; Tolinacki, M; Golic, N; Kataranovski, M | 2019 | Pulmonary Aspergillus fumigatus infection in rats affects gastrointestinal homeostasis | **Eukaryote + Prokaryote** | 16S + ITS + 18S | Rats | <http://dx.doi.org/10.1016/j.imbio.2018.10.001> |
| 163 | | Zhu, WH; Winter, MG; Byndloss, MX; Spiga, L; Duerkop, BA; Hughes, ER; Buttner, L; Romao, ED; Behrendt, CL; Lopez, CA; Sifuentes-Dominguez, L; Huff-Hardy, K; Wilson, RP; Gillis, CC; Tukel, C; Koh, AY; Burstein, E; Hooper, LV; Baumler, AJ; Winter, SE | 2018 | Precision editing of the gut microbiota ameliorates colitis | **Prokaryote** | 16S | Mice | <http://dx.doi.org/10.1038/nature25172> |
| 164 | | Wang, W; Wang, AZ; Yang, YS; Wang, F; Liu, YB; Zhang, YH; Sharshov, K; Gui, LS | 2019 | Composition, diversity and function of gastrointestinal microbiota in wild red-billed choughs (Pyrrhocorax pyrrhocorax) | **Prokaryote** | 16S | Birds | <http://dx.doi.org/10.1007/s10123-019-00076-2> |
| 165 | | Bains, M; Laney, C; Wolfe, AE; Orr, M; Waschek, JA; Ericsson, AC; Dorsam, GP | 2019 | Vasoactive Intestinal Peptide Deficiency Is Associated With Altered Gut Microbiota Communities in Male and Female C57BL/6 Mice | **Prokaryote** | 16S | Mice | <http://dx.doi.org/10.3389/fmicb.2019.02689> |
| 166 | | Ding, H; Zhao, X; Ma, C; Gao, Q; Yin, Y; Kong, X; He, J | 2021 | Dietary supplementation withBacillus subtilisDSM 32315 alters the intestinal microbiota and metabolites in weaned piglets | **Prokaryote** | 16S | pigs | <http://dx.doi.org/10.1111/jam.14767> |
| 167 | | Kjolbaek, L; Benitez-Paez, A; del Pulgar, EMG; Brahe, LK; Liebisch, G; Matysik, S; Rampelli, S; Vermeiren, J; Brigidi, P; Larsen, LH; Astrup, A; Sanz, Y | 2020 | Arabinoxylan oligosaccharides and polyunsaturated fatty acid effects on gut microbiota and metabolic markers in overweight individuals with signs of metabolic syndrome: A randomized cross-over trial | **Prokaryote** | 16S | Human | <http://dx.doi.org/10.1016/j.clnu.2019.01.012> |
| 168 | | Hale, VL; Tan, CL; Niu, KF; Yang, YQ; Zhang, QK; Knight, R; Amato, KR | 2019 | Gut microbiota in wild and captive Guizhou snub-nosed monkeys, Rhinopithecus brelichi | **Prokaryote** | 16S | Monkey | <http://dx.doi.org/10.1002/ajp.22989> |
| 169 | | Park, T; Cheong, H; Yoon, J; Kim, A; Yun, YM; Unno, T | 2021 | Comparison of the Fecal Microbiota of Horses with Intestinal Disease and Their Healthy Counterparts | **Prokaryote** | 16S | Horses | <http://dx.doi.org/10.3390/vetsci8060113> |
| 170 | | Fogarty, C; Burgess, CM; Cotter, PD; Cabrera-Rubio, R; Whyte, P; Smyth, C; Bolton, DJ | 2019 | Diversity and composition of the gut microbiota of Atlantic salmon (Salmo salar) farmed in Irish waters | **Prokaryote** | 16S | Fish | <http://dx.doi.org/10.1111/jam.14291> |
| 171 | | Yamamoto, Y; Nakanishi, Y; Murakami, S; Aw, WP; Tsukimi, T; Nozu, R; Ueno, M; Hioki, K; Nakahigashi, K; Hirayama, A; Sugimoto, M; Soga, T; Ito, M; Tomita, M; Fukuda, S | 2018 | A Metabolomic-Based Evaluation of the Role of Commensal Microbiota throughout the Gastrointestinal Tract in Mice | **Prokaryote** | 16S | Mice | <http://dx.doi.org/10.3390/microorganisms6040101> |
| 172 | | Ellermann, M; Gharaibeh, RZ; Maharshak, N; Perez-Chanona, E; Jobin, C; Carroll, IM; Arthur, JC; Plevy, SE; Fodor, AA; Brouwer, CR; Sartor, RB | 2020 | Dietary iron variably modulates assembly of the intestinal microbiota in colitis-resistant and colitis-susceptible mice | **Prokaryote** | 16S | Mice | <http://dx.doi.org/10.1080/19490976.2019.1599794> |
| 173 | | Zhou, QQ; Lan, FR; Li, XC; Yan, W; Sun, CJ; Li, JY; Yang, N; Wen, CL | 2021 | The Spatial and Temporal Characterization of Gut Microbiota in Broilers | **Prokaryote** | 16S | Birds | <http://dx.doi.org/10.3389/fvets.2021.712226> |
| 174 | | Guo, YY; Xu, M; Shi, GZ; Zhang, JD | 2021 | A new strategy of enteral nutrition intervention for ICU patients targeting intestinal flora | **Eukaryote + Prokaryote** | 16S + 18S | Human | <http://dx.doi.org/10.1097/MD.0000000000027763> |
| 175 | | Wang, JY; Xiong, K; Zhao, SL; Zhang, C; Zhang, JW; Xu, L; Ma, AG | 2020 | Long-Term Effects of Multi-Drug-Resistant Tuberculosis Treatment on Gut Microbiota and Its Health Consequences | **Prokaryote** | 16S | Human | <http://dx.doi.org/10.3389/fmicb.2020.00053> |
| 176 | | Qian, LM; Gao, RY; Hong, LM; Pan, C; Li, H; Huang, JM; Qin, HL | 2018 | Association analysis of dietary habits with gut microbiota of a native Chinese community | **Prokaryote** | 16S | Human | <http://dx.doi.org/10.3892/etm.2018.6249> |
| 177 | | Viso, NP; Redondo, E; Carrasco, JMD; Redondo, L; Garcia, JSY; Miyakawa, MF; Farber, MD | 2021 | Geography as non-genetic modulation factor of chicken cecal microbiota | **Prokaryote** | 16S | Birds | <http://dx.doi.org/10.1371/journal.pone.0244724> |
| 178 | | Li, Y; Liu, M; Zhou, J; Hou, B; Su, X; Liu, Z; Yuan, J; Li, M | 2019 | Bacillus licheniformis Zhengchangsheng (R) attenuates DSS-induced colitis and modulates the gut microbiota in mice | **Prokaryote** | 16S | Mice | <http://dx.doi.org/10.3920/BM2018.0122> |
| 179 | | Cao, H; Li, CN; Lei, L; Wang, X; Liu, SN; Liu, Q; Huan, Y; Sun, SJ; Shen, ZF | 2020 | Stachyose Improves the Effects of Berberine on Glucose Metabolism by Regulating Intestinal Microbiota and Short-Chain Fatty Acids in Spontaneous Type 2 Diabetic KKAy Mice | **Prokaryote** | 16S | Mice | <http://dx.doi.org/10.3389/fphar.2020.578943> |
| 180 | | Ding, X; Xu, YR; Zhang, XL; Zhang, LL; Duan, GQ; Song, CL; Li, ZH; Yang, YY; Wang, YZ; Wang, XY; Zhu, CL | 2020 | Gut microbiota changes in patients with autism spectrum disorders | **Prokaryote** | 16S | Human | <http://dx.doi.org/10.1016/j.jpsychires.2020.06.032> |
| 181 | | Lin, H; Meng, LP; Sun, ZZ; Sun, SM; Huang, XX; Lin, N; Zhang, J; Lu, WQ; Yang, Q; Chi, JF; Guo, HY | 2021 | Yellow Wine Polyphenolic Compound Protects Against Doxorubicin-Induced Cardiotoxicity by Modulating the Composition and Metabolic Function of the Gut Microbiota | **Prokaryote** | 16S | Rats | <http://dx.doi.org/10.1161/CIRCHEARTFAILURE.120.008220> |
| 182 | | Xiong, JJ; Hu, HW; Xu, CZ; Yin, JW; Liu, M; Zhang, LZ; Duan, Y; Huang, YK | 2022 | Development of gut microbiota along with its metabolites of preschool children | **Prokaryote** | 16S | Human | <http://dx.doi.org/10.1186/s12887-021-03099-9> |
| 183 | | Huang, CF; Li, XY; Wu, LP; Wu, GF; Wang, PQ; Peng, YP; Huang, SY; Yang, ZY; Dai, WK; Ge, L; Lyu, YS; Wang, LL; Zhang, AQ | 2021 | The effect of different dietary structure on gastrointestinal dysfunction in children with cerebral palsy and epilepsy based on gut microbiota | **Prokaryote** | 16S | Human | <http://dx.doi.org/10.1016/j.braindev.2020.09.013> |
| 184 | | Fan, QJ; Yi, M; Liu, H; Wang, YS; Li, XK; Yuan, JL; Wang, LL; Hou, BB; Li, M | 2020 | The Impact of Age and Pathogens Type on the Gut Microbiota in Infants with Diarrhea in Dalian, China | **Prokaryote** | 16S | Human | <http://dx.doi.org/10.1155/2020/8837156> |
| 185 | | Kortekangas, E; Young, R; Cheung, YB; Fan, YM; Jorgensen, JM; Kamng'ona, AW; Chaima, D; Ashorn, U; Dewey, KG; Maleta, K; Ashorn, P | 2019 | A Prospective Study on Child Morbidity and Gut Microbiota in Rural Malawi | **Prokaryote** | 16S | Human | <http://dx.doi.org/10.1097/MPG.0000000000002435> |
| 186 | | Natalello, G; Bosello, SL; Sterbini, FP; Posteraro, B; De Lorenzis, E; Canestrari, GB; Gigante, L; Verardi, L; Ferraccioli, G; Sanguinetti, M; Gremese, E | 2020 | Gut microbiota analysis in systemic sclerosis according to disease characteristics and nutritional status | **Prokaryote** | 16S | Human | <https://pubmed.ncbi.nlm.nih.gov/32865168/> |
| 187 | | Li, XX; Shi, S; Rong, L; Feng, MQ; Zhong, L | 2018 | The impact of liposomal linolenic acid on gastrointestinal microbiota in mice | **Prokaryote** | 16S | Mice | <http://dx.doi.org/10.2147/IJN.S151825> |
| 188 | | Piazzon, MC; Naya-Catala, F; Simo-Mirabet, P; Picard-Sanchez, A; Roig, FJ; Calduch-Giner, JA; Sitja-Bobadilla, A; Perez-Sanchez, J | 2019 | Sex, Age, and Bacteria: How the Intestinal Microbiota Is Modulated in a Protandrous Hermaphrodite Fish | **Prokaryote** | 16S | Fish | <http://dx.doi.org/10.3389/fmicb.2019.02512> |
| 189 | | Liebisch, G; Plagge, J; Horing, M; Seeliger, C; Ecker, J | 2021 | The effect of gut microbiota on the intestinal lipidome of mice | **Prokaryote** | 16S | Mice | <http://dx.doi.org/10.1016/j.ijmm.2021.151488> |
| 190 | | Stolzenbach, S; Myhill, LJ; Andersen, LO; Krych, L; Mejer, H; Williams, AR; Nejsum, P; Stensvold, CR; Nielsen, DS; Thamsborg, SM | 2020 | Dietary Inulin and Trichuris suis Infection Promote Beneficial Bacteria Throughout the Porcine Gut | **Prokaryote** | 16S | Pigs | <http://dx.doi.org/10.3389/fmicb.2020.00312> |
| 191 | | Tang, WJ; Zhu, GX; Shi, Q; Yang, S; Ma, TY; Mishra, SK; Wen, AX; Xu, HL; Wang, Q; Jiang, YZ; Wu, JY; Xie, M; Yao, YF; Li, DY | 2019 | Characterizing the microbiota in gastrointestinal tract segments of Rhabdophis subminiatus: Dynamic changes and functional predictions | **Prokaryote** | 16S | Snake | <http://dx.doi.org/10.1002/mbo3.789> |
| 192 | | Li, B; Li, M; Luo, YN; Li, R; Li, W; Liu, Z | 2022 | Engineered 5-HT producing gut probiotic improves gastrointestinal motility and behavior disorder | **Prokaryote** | 16S | Mice | <http://dx.doi.org/10.3389/fcimb.2022.1013952> |
| 193 | | Wang, TW; Teng, KL; Liu, G; Liu, YY; Zhang, J; Zhang, X; Zhang, M; Tao, Y; Zhong, J | 2018 | Lactobacillus reuteri HCM2 protects mice against Enterotoxigenic Escherichia coli through modulation of gut microbiota | **Prokaryote** | 16S | Mice | <http://dx.doi.org/10.1038/s41598-018-35702-y> |
| 194 | | Liu, Y; Wang, T; Si, B; Du, H; Liu, Y; Waqas, A; Huang, SW; Zhao, GP; Chen, SP; Xu, A | 2021 | Intratracheally instillated diesel PM2.5 significantly altered the structure and composition of indigenous murine gut microbiota | **Prokaryote** | 16S | Mice | <http://dx.doi.org/10.1016/j.ecoenv.2021.111903> |
| 195 | | Wu, DT; Yuan, Q; Guo, H; Fu, Y; Li, F; Wang, SP; Gan, RY | 2021 | Dynamic changes of structural characteristics of snow chrysanthemum polysaccharides during in vitro digestion and fecal fermentation and related impacts on gut microbiota | **Prokaryote** | 16S | in vitro | <http://dx.doi.org/10.1016/j.foodres.2020.109888> |
| 196 | | Son, M; Park, IS; Kim, S; Ma, HW; Kim, JH; Kim, TI; Kim, WH; Han, J; Kim, SW; Cheon, JH | 2022 | Novel Potassium-Competitive Acid Blocker, Tegoprazan, Protects Against Colitis by Improving Gut Barrier Function | **Prokaryote** | 16S | Mice | <http://dx.doi.org/10.3389/fimmu.2022.870817> |
| 197 | | Qi, XF; Li, XW; Zhao, Y; Wu, XJ; Chen, F; Ma, X; Zhang, FM; Wu, DP | 2018 | Treating Steroid Refractory Intestinal Acute Graft-vs.-Host Disease With Fecal Microbiota Transplantation: A Pilot Study | **Prokaryote** | 16S | Human | <http://dx.doi.org/10.3389/fimmu.2018.02195> |
| 198 | | Jing, YL; Yang, DG; Bai, F; Zhang, C; Qin, C; Li, D; Wang, LM; Yang, ML; Chen, ZG; Li, JJ | 2019 | Melatonin Treatment Alleviates Spinal Cord Injury-Induced Gut Dysbiosis in Mice | **Prokaryote** | 16S | Mice | <http://dx.doi.org/10.1089/neu.2018.6012> |
| 199 | | Babbar, A; Hitch, TCA; Pabst, O; Clavel, T; Hubel, J; Eswaran, S; Wagner, N; Schippers, A | 2019 | The Compromised Mucosal Immune System of beta 7 Integrin-Deficient Mice Has Only Minor Effects on the Fecal Microbiota in Homeostasis | **Prokaryote** | 16S | Mice | <http://dx.doi.org/10.3389/fmicb.2019.02284> |
| 200 | | Fu, YS; Zhang, JN; Chen, KN; Xiao, CX; Fan, LN; Zhang, BZ; Ren, JL; Fang, BS | 2019 | An in vitro fermentation study on the effects of Dendrobium officinale polysaccharides on human intestinal microbiota from fecal microbiota transplantation donors | **Prokaryote** | 16S | in vitro | <http://dx.doi.org/10.1016/j.jff.2018.12.005> |
| 201 | | Tang, DZ; Zeng, T; Wang, YT; Cui, H; Wu, JY; Zou, B; Tao, ZD; Zhang, L; Garside, GB; Tao, S | 2020 | Dietary restriction increases protective gut bacteria to rescue lethal methotrexate-induced intestinal toxicity | **Prokaryote** | 16S | Mice | <http://dx.doi.org/10.1080/19490976.2020.1714401> |
| 202 | | Sun, XZ; Wang, DD; Wei, LA; Ding, LZ; Guo, YA; Wang, ZT; Kong, YB; Yang, JJ; Sun, LW; Sun, LP | 2021 | Gut Microbiota and SCFAs Play Key Roles in QingFei Yin Recipe Anti-Streptococcal Pneumonia Effects | **Prokaryote** | 16S | Mice | <http://dx.doi.org/10.3389/fcimb.2021.791466> |
| 203 | | Kullberg, RFJ; Haak, BW; Abdel-Aziz, MI; Davids, M; Hugenholtz, F; Nieuwdorp, M; Galenkamp, H; Prins, M; Maitland-van der Zee, AH; Wiersinga, WJ | 2021 | Gut microbiota of adults with asthma is broadly similar to non-asthmatics in a large population with varied ethnic origins | **Prokaryote** | 16S | Human | <http://dx.doi.org/10.1080/19490976.2021.1995279> |
| 204 | | Gonzales, J; Marchix, J; Aymeric, L; Le Berre-Scoul, C; Zoppi, J; Bordron, P; Burel, M; Davidovic, L; Richard, JR; Gaman, A; Lejuste, F; Brouillet, JZ; Le Vacon, F; Chaffron, S; Leboyer, M; Boudin, H; Neunlist, M | 2021 | Fecal Supernatant from Adult with Autism Spectrum Disorder Alters Digestive Functions, Intestinal Epithelial Barrier, and Enteric Nervous System | **Prokaryote** | 16S | Human | <http://dx.doi.org/10.3390/microorganisms9081723> |
| 205 | | Sanchez-Leon, S; Haro, C; Villatoro, M; Vaquero, L; Comino, I; Gonzalez-Amigo, AB; Vivas, S; Pastor, J; Sousa, C; Landa, BB; Barro, F | 2021 | Tritordeum breads are well tolerated with preference over gluten-free breads in non-celiac wheat-sensitive patients and its consumption induce changes in gut bacteria | **Prokaryote** | 16S | Human | <http://dx.doi.org/10.1002/jsfa.10982> |
| 206 | | Williams, T; Athrey, G | 2020 | Cloacal Swabs Are Unreliable Sources for Estimating Lower Gastro-Intestinal Tract Microbiota Membership and Structure in Broiler Chickens | **Prokaryote** | 16S | Birds | <http://dx.doi.org/10.3390/microorganisms8050718> |
| 207 | | Ji, F; Zhang, DY; Shao, YX; Yu, XH; Liu, XY; Shan, DC; Wang, Z | 2020 | Changes in the diversity and composition of gut microbiota in pigeon squabs infected with Trichomonas gallinae | **Prokaryote** | 16S | Birds | <http://dx.doi.org/10.1038/s41598-020-76821-9> |
| 208 | | Liang, WR; Yang, Y; Wang, HY; Wang, HH; Yu, XF; Lu, YM; Shen, SR; Teng, LS | 2019 | Gut microbiota shifts in patients with gastric cancer in perioperative period | **Prokaryote** | 16S | Human | <http://dx.doi.org/10.1097/MD.0000000000016626> |
| 209 | | Grayson, MH; Camarda, LE; Hussain, SRA; Zemple, SJ; Hayward, M; Lam, V; Hunter, DA; Santoro, JL; Rohlfing, M; Cheung, DS; Salzman, NH | 2018 | Intestinal Microbiota Disruption Reduces Regulatory T Cells and Increases Respiratory Viral Infection Mortality Through Increased IFN gamma Production | **Prokaryote** | 16S | Mice | <http://dx.doi.org/10.3389/fimmu.2018.01587> |
| 210 | | Lee, WT; Tung, YT; Wu, CC; Tu, PS; Yen, GC | 2018 | Camellia Oil (Camellia oleifera Abel.) Modifies the Composition of Gut Microbiota and Alleviates Acetic Acid-Induced Colitis in Rats | **Prokaryote** | 16S | Rats | <http://dx.doi.org/10.1021/acs.jafc.8b02166> |

**2. Supplementary Figures**

**Figure S1** Percentage results of scientific research grouped according to the marker gene used, derived from the ISI Web of Knowledge platform search with a pre-established search algorithm (gut microbiota OR intestinal microbiota OR gastrointestinal microbiota).


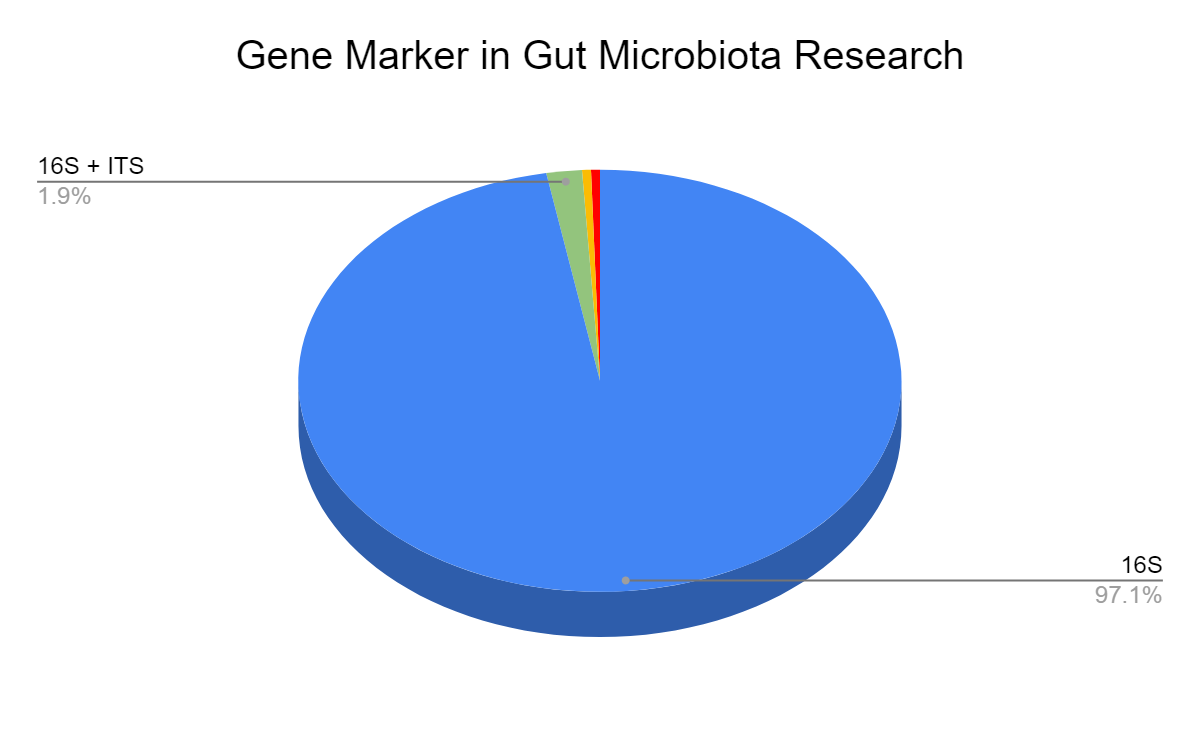


| **Marker gen** | **Research** |
| --- | --- |
| 16S | 204 |
| 16S + ITS | 4 |
| 16S + ITS + 18S | 1 |
| 16S + 18S | 1 |
| 18S | 0 |
